# Neutrophil extracellular traps (NETs) are increased in rheumatoid arthritis-associated interstitial lung disease

**DOI:** 10.1186/s12931-025-03111-1

**Published:** 2025-01-22

**Authors:** Jing Xue, Miaomiao Nian, Yangyang Liang, Zeqin Zhu, Zhenyu Hu, Yuanyuan Jia, Shuhong Chi, Juan Chen

**Affiliations:** 1 Department of Key Laboratory of Ningxia Stem Cell and Regenerative Medicine, Institute of Medical Sciences, General Hospital of Ningxia Medical University, Yinchuan, 750004 Ningxia China; 2 Ningxia Medical University, Yinchuan, 750004 Ningxia China; 3 Department of Rheumatology, General Hospital of Ningxia Medical University, Yinchuan, 750004 Ningxia China; 4 Department of Pulmonary and Critical Care Medicine, General Hospital of Ningxia Medical University, Yinchuan, 750004 Ningxia China

**Keywords:** Nuclear receptor 4, Neutrophil extracellular traps, Rheumatoid arthritis, Interstitial lung disease

## Abstract

**Background:**

Neutrophil extracellular trap (NET) formation has been implicated as a pathogenic mechanism in both rheumatoid arthritis (RA) and interstitial lung disease (ILD). However, the role of NETs in RA-associated ILD (RA-ILD) and the mechanisms driving NET formation remain unclear. This study aimed to assess the involvement of NETs in RA-ILD and elucidate the underlying mechanisms.

**Methods:**

Single-cell sequencing was used to identify changes in the quantity and function of neutrophils in the lung tissue of a zymosan A (ZYM)-induced interstitial pneumonia arthritis model. Additionally, nuclear receptor 4A3 (NR4A3) interference was performed in HL-60 cells to assess its impact on NET formation and the transformation of MRC-5 cells into myofibroblasts. The clinical relevance of plasma myeloperoxidase-DNA (MPO-DNA), citrullinated histone 3 (Cit-H3), and cell-free DNA was evaluated in RA-ILD patients with different imaging types via a commercial enzyme-linked immunosorbent assay (ELISA).

**Results:**

In the ZYM-treated SKG mouse model, which recapitulates key features of RA-ILD, an increased population of neutrophils in the lung tissue was primarily responsible for NET formation. Mechanistically, we found that interference with NR4A3 expression enhanced NET formation in HL-60 cells, which in turn promoted the differentiation of MRC-5 cells into myofibroblasts. Clinically, plasma MPO-DNA levels are elevated in patients with RA-nonspecific interstitial pneumonia (RA-NSIP), whereas Cit-H3 levels are elevated in RA-usual interstitial pneumonia (RA-UIP) patients compared with healthy subjects. ROC curve analysis further revealed that the combination of plasma MPO-DNA, rheumatoid factor (RF), and anti-citrullinated protein (anti-CCP) and the combination of Cit-H3, RF, and anti-CCP were superior diagnostic panels for NSIP and UIP in RA-ILD patients, respectively. Moreover, compared with those from healthy controls, neutrophils from patients with RA-UIP and RA-NSIP demonstrated a significantly increased ability to form NETs and induce the differentiation of MRC-5 cells into myofibroblasts. Specifically, RA-UIP patients exhibited a greater capacity for NET formation and the differentiation of MRC-5 cells into myofibroblasts than did RA-NSIP patients.

**Conclusions:**

These findings suggest that targeting NETs may be a novel therapeutic approach for treating ILD in RA patients.

**Supplementary Information:**

The online version contains supplementary material available at 10.1186/s12931-025-03111-1.

## Introduction

Interstitial lung disease (ILD) is a prevalent but frequently underrecognized complication of rheumatoid arthritis (RA) and significantly impairs patient outcomes [1, 2]. The survival rate of patients with RA-ILD has been reported to be comparable to that of patients with idiopathic pulmonary fibrosis (IPF), primarily due to its progressive nature and the limited availability of effective therapeutic options [3, 4]. Despite the substantial impact on patient prognosis, the underlying pathophysiological mechanisms remain poorly understood. Therefore, elucidating the molecular pathways that drive RA-ILD is crucial for identifying novel therapeutic targets.

ILD represents a diverse group of pulmonary fibrotic and inflammatory conditions [5]. Although the mechanisms underlying the dysregulation of inflammatory cascades and tissue remodeling in various forms of RA-ILD remain obscure, accumulating evidence suggests that neutrophils play a pivotal role in the complex interplay between inflammation and fibrosis in this context [6, 7]. For example, neutrophils produce tissue inhibitors of metalloproteinases and neutrophil elastase (NE), which together activate transforming growth factor β (TGF-β) and recruit other inflammatory cells to the lungs, promoting pulmonary fibrosis [6]. Additionally, neutrophils release neutrophil extracellular traps (NETs), which contain chromatin bound to granule enzymes and are implicated in both inflammation and fibrosis [6]. NETs are large, web-like structures that consist of of cytosolic and granule proteins assembled on a scaffold of decondensed chromatin that can accumulate in the lungs and exacerbate fibrosis [8]. The formation of NETs is a complex process that involves multiple molecular mechanisms and signaling pathways. Suicidal NET formation, induced by phorbol 12-myristate 13-acetate (PMA), immune complexes, or bacteria, activates NADPH oxidase (NOX) to produce reactive oxygen species (ROS), which subsequently activate myeloperoxidase (MPO) and neutrophil elastase (NE), leading to chromatin decondensation and eventual neutrophil death. In contrast, vital NET formation is triggered by factors such as *platelets* or *Staphylococcus aureus*, which involve the release of nuclear DNA to form NETs while maintaining the antibacterial and secretory functions of neutrophils. Mitochondrial NET formation, driven by granulocyte‒macrophage colony‒stimulating factor (GM-CSF) combined with lipopolysaccharide/complement C5a (LPS/C5a), leads to the release of mitochondrial DNA to form NETs, with neutrophils maintaining their physiological activity. Additionally, peptidylarginine deiminase 4 (PADI4) is activated by calcium ion channels and then translocates to the nucleus to mediate histone citrullination and chromatin decondensation [9].

Recent studies have highlighted the contribution of NETs to the pathogenesis of immune-related diseases, including RA [10] and dermatomyositis (DM)-associated ILD [11]. Pérez-Sánchez et al. reported that 6 months of therapy with tocilizumab (TCZ) or infliximab not only improved disease activity but also reduced the release of extracellular DNA by decreasing NET formation in RA patients [12]. Furthermore, the development of drugs that target NET formation represents a promising therapeutic strategy for the treatment of RA [13]. In the context of ILD, patients with antimelanoma differentiation-associated gene 5 autoantibody-positive (MDA5 Ab^+^) dermatomyositis (DM) have higher concentrations of serum circulating free DNA than patients with anti-MDA5 Ab^−^ DM [14]. Taken together, accumulating evidence suggests that NETs may contribute to the development of RA-ILD.

Nuclear receptor 4A3 (NR4A3), also known as neuron-derived orphan receptor 1 (NOR-1), is a member of the NR4A orphan nuclear receptor subfamily [15]. Although NR4A3 is expressed in various cell types, its functional role remains poorly understood. Several studies have shown that NR4A3 is upregulated in cultured Ins-1 cells and human pancreatic islets treated with IL-1β and TNF-α. Interestingly, NR4A3 knockdown reduces cytokine-mediated apoptosis, whereas NR4A3 overexpression enhances apoptotic cell death via proinflammatory pathways [16]. These findings indicate that NR4A3 plays a proapoptotic role. Moreover, the downregulation of NR4A3 leads to a reduction in the transcription of *Sdha*, which significantly impairs mitochondrial ATP production, increases oxidative stress, and contributes to atrial hypertrophy, fibrosis, and electrical remodeling [17]. Notably, elevated oxidative stress levels are also associated with the excessive formation of NETs in inflammatory and autoimmune diseases [18]. These data suggest that NR4A3 may be involved in regulating NET formation.

Hence, the primary objective of this study was to investigate the correlation between NETs and RA-ILD, explore the underlying mechanisms driving NET formation, and assess the clinical implications of these findings.

## Materials and methods

### Animal welfare statement and generation of a zymosan A-induced interstitial pneumonia arthritic mouse model

All mouse protocols were approved by the Laboratory Animal Committee of Ningxia University in accordance with the guidelines of the National Institutes of Health Guide for the Care and Use of Laboratory Animals (KYLL-2024-1033). All the mice were purchased from CLEA Japan Inc. (Tokyo, Japan) and housed in a special pathogen-free facility at the animal facility of Ningxia Medical University (Yinchuan, China) with a 12/12-hour light/dark cycle, with food and water *ad libitum*. To generate an arthritic mouse model of interstitial pneumonia, 8-week-old SKG/Jcl mice (*n* = 12) were intraperitoneally administered 7.5 mg of zymosan A (ZYM) (Alfa Aesar, Lancashire, UK) dissolved in 0.5 mL of phosphate-buffered saline (PBS). The control mice (*n* = 12) were administered 0.5 mL of PBS. Body weight and paw size were examined weekly. The arthritis score for each of the four paws was recorded via the following scoring system: 0 = normal joints; 1 = slight swelling or erythema of the ankle or midfoot; 2 = slight swelling of the ankle and foot; 3 = moderate swelling and erythema; and 4 = severe swelling and erythema [19]. The maximum score was 16 for each mouse. The mice were euthanized via carbon dioxide (CO_2_) inhalation at the end of the experiment. Tissues, including blood, knee joints, brain, heart, kidney, liver, spleen, colon, and lungs, were harvested for pathological and molecular analysis at 8 and 16 weeks post-ZYM challenge.

### Lung and joint histopathological analysis

Mouse lung tissues were fixed in 4% paraformaldehyde (PFA) solution before being embedded in optimal cutting temperature (OCT) compound or dehydrated and processed for paraffin embedding. Five-micrometer-thick sections were prepared and stained using hematoxylin and eosin (H&E), Masson’s trichrome, or other methods as described elsewhere [20]. The severity of interstitial injury and fibrosis in lung tissues was assessed by evaluating inflammatory cell infiltration using the Ashcroft scale, with scores ranging from 0 to 4 for interstitial injury and 0 to 8 for fibrosis, based on histological images of the lung as previously described [21].

Joint tissues were fixed in 10% formalin, decalcified with EDTA, embedded in paraffin, and cut into five-micrometer-thick sections. H&E and toluidine blue-O (TBO) staining were performed. The degree of joint inflammation and destruction was assessed by evaluating inflammatory cell infiltration and cartilage damage, and both were scored by two independent, blinded pathologists. Both joint inflammation and joint destruction were evaluated on a scale of 0–4, as previously described [22].

### Measuring anti-CCP, RF, histone H3, cell-free DNA, and MPO-DNA concentrations

The concentrations of anti-citrullinated protein (anti-CCP) antibodies, RF, MPO-DNA, citrullinated histone H3 (Cit-H3), and cell-free DNA were measured using commercially available enzyme-linked immunosorbent assay (ELISA) kits following the manufacturers’ instructions. ELISA kits for detecting mouse anti-CCP and RF were purchased from Shanghai JiangLai Biotechnology Co., Ltd. (Shanghai, China). The kits for detecting MPO-DNA, Cit-H3, and cell-free DNA were obtained from Shanghai HengYuan Biotechnology Co., Ltd. (Shanghai, China). The protein concentration in each sample was determined via comparison with a standard curve.

### Micro-CT scanning evaluation

At 16 weeks post injection, the knee joints of the mice were harvested and fixed in 4% paraformaldehyde. The samples were scanned via micro-CT (µCT 40; Scanco, Zurich, Switzerland) as previously described [23]. The scanner was set to a resolution of 10 μm with a voltage of 70 kV and an electric current of 114 µA. The region of interest (ROI) was defined to cover the entire subchondral bone in the tibial plateaus. The three-dimensional structural parameters analyzed included the bone volume (BV), bone volume/total tissue volume (BV/TV), trabecular thickness (Tb.Th), and trabecular separation (Tb.Sp).

### RNA isolation and RT‒PCR

Total RNA was isolated using the MiniBEST Universal RNA Extraction Kit (TaKaRa) following the manufacturer’s instructions. Quantitative RT‒PCR with SYBR Green Master Mix (TaKaRa, Dalian, China) was performed using the StepOnePlus RT‒PCR System. The relative expression levels of the target mRNAs were quantified via the delta‒delta Ct method, with the primer sequences provided in Supplementary Table S1.

### Western blotting

Proteins were extracted from both cell lines and tissues via RIPA lysis buffer (Thermo Fisher Science, USA) under cold conditions. After centrifugation at 12,000 × g for 10 min, protein concentrations were determined using a BCA protein assay kit (Thermo Fisher Science, USA). Protein samples were loaded onto PAGE gels (Epizyme Biomedical Technology, Shanghai, China) and transferred to 0.22 μm Immobilon PVDF membranes (Millipore Sigma, USA). Following blocking with 5% milk, the membranes were incubated with primary antibodies at appropriate dilutions overnight at 4 °C. The secondary antibodies were applied at room temperature for 1 h, and the immunoreactivity was visualized using an ECL system (Thermo Fisher Scientific, Waltham, MA). Relative protein expression was determined semiquantitatively via densitometric analysis of the blots. The intensity of each blot area was measured via ImageJ Software version 2.0.0 (http://rsb.info.nib.gov/ij). The ratio of the net intensity of each sample to that of the housekeeping gene GAPDH served as an internal loading control. The values are reported as arbitrary densitometric units (A.U.). The relative target protein expression in the experimental group was normalized to that in the control group to calculate the fold change. The primary and secondary antibodies used for immunoblotting are listed in Supplementary Table S2.

### Immunofluorescence staining

Fixed lung tissues embedded in OCT compound were cryosectioned at a thickness of 8 μm for immunofluorescence (IF). The cryosections were air dried at RT for 30 min, fixed in 4% PFA for 10 min, and permeabilized in 0.2% Triton X-100/PBS for 20 min at RT. The sections were blocked by incubation in 5% donkey serum in PBS for 1 h before being probed with primary antibodies in diluent buffer (1% donkey serum, 0.03% Triton X-100, and 1 mM CaCl_2_ in PBS) overnight at 4 °C. The sections were washed and then incubated with fluorescent dye-conjugated secondary antibodies at RT for 2 h. The slides were washed in PBS three times for 5 min, mounted with VECTASHIELD Antifade Mounting Medium with DAPI (Vector Laboratories), and imaged with a Leica TCS SP2 AOBS confocal system. Images were processed using Leica Confocal Software v.2.6.1 (Leica). The primary and secondary antibodies used for IF are listed in Supplementary Table S2.

### Immunohistochemistry

Paraffin-embedded tissue samples were sectioned, deparaffinized with xylene, and rehydrated in decreasing concentrations of ethanol, followed by 3 washes with 1× PBS. Antigen retrieval was performed in boiling sodium citrate buffer (10 mmol/L sodium citrate, 0.05% Tween-20, pH 6.0) or with Proteinase-K (20 µg/mL). The sections were then blocked with 3% fetal bovine serum in 1× PBS for 30 min, followed by overnight incubation at 4 °C. After 3 washes with 1× PBS, the sections were further incubated with biotin- or HRP-conjugated secondary antibodies for 30 min at RT. Antibody staining was developed with DAB substrate (Zsbio, Beijing, China) for HRP-conjugated secondary antibodies or incubated with an avidin-biotin complex staining kit (ABC Kit; Vector Laboratories, Burlingame, CA) before DAB development, following the manufacturer’s instructions. The primary and secondary antibodies used for IHC are listed in Supplementary Table S2.

### Preparation of single-cell suspensions

Single-cell suspensions were prepared according to previously established protocols [24]. Briefly, lung lobes from control and ZYM-treated mice were digested with collagenase type IA and deoxyribonuclease I (Thermo Fisher Scientific Inc., Waltham, MA, USA), followed by trypsinization. The cell suspension was then filtered through a 40 μm cell strainer, washed, centrifuged, and finally resuspended in magnetic-activated cell sorting buffer (Miltenyi Biotec, Bergisch Gladbach, North Rhine-Westphalia, Germany) for single-cell RNA sequencing (scRNA-Seq) analysis.

### Construction of single-cell libraries and sequencing

In brief, single-cell suspensions and beads were mixed by adjusting the coencapsulation occupancy to 0.05. After individual droplets were collected, messenger RNA was reverse transcribed into complementary DNA, followed by cDNA amplification. Finally, a 3’gene expression library was prepared via the Chromium Next GEM Single Cell 3’Kit v3.1 (10× Genomics, Pleasanton, CA, USA). Sequencing was conducted by using a NovaSeq 6,000 (Illumina, San Diego, CA, USA) by OE Biotech Co., Ltd. (Shanghai, China).

### ScRNA-seq data quality control analysis

Raw sequencing reads of mouse lung tissues were aligned to the mouse genome reference (GENCODE, mm10) and processed into a single-cell matrix via Cell Ranger (version 7.0.0) with the default parameters. Considering the presence of double droplets, empty droplets, dead cells, low-quality cells with nFeature_RNA > 4000, percent.mt > 10 and nFeature_RNA < 1000 were excluded. A total of 37,735 cells were finally identified after filtration in the dataset. The data were subsequently normalized, the dimensionality was reduced, and cell clustering was performed via Seurat (v.4.1.1) [24]. Additionally, the clusters with relatively low gene numbers and the absence of specific marker genes were also removed.

### Integration and cell type annotation

To integrate each sample and correct for batch effects, the top 2000 highly variable genes (HVGs) were identified via the Seurat function FindVariableGenes (mean.function = FastExpMean, dispersion.function = FastLogVMR). To remove batch effects from the ScRNA-Seq data, the mutual nearest neighbors (MNN) method presented by Haghverdi et al. was performed with the R package batchelor (version 1.6.3) [25]. Graph-based clustering was performed to cluster cells according to their gene expression profile with the FindClusters function. The cells were visualized via a 2-dimensional uniform manifold approximation and projection (UMAP) algorithm with the RunUMAP function. Finally, the FindAllMarkers function (test.use = presto) was used to identify marker genes of each cluster.

### Differentially expressed gene analysis

Differentially expressed genes (DEGs) were selected via the function FindMarkers (test.use = presto). A *P* value < 0.05 and |log2fold change|>0.58 were set as the thresholds for significantly differential expression.

### Gene ontology enrichment analysis

Gene Ontology (GO) enrichment and Kyoto Encyclopedia of Genes and Genomes (KEGG) pathway enrichment analyses of the DEGs were conducted via R (version 4.0.3), and the hypergeometric distribution was used as the statistical method.

### Microarray data and identification of hub genes

The transcription profile dataset of lung tissues in the RA-UIP, IPF-UIP and non-UIP control groups (GSE199152) was obtained from the NCBI GEO database (http://www.ncbi.nlm.nih.gov/geo/). The platform is GPL16791 and includes 27 subjects: 3 RA-UIP patients, 20 IPF patients, and 4 non-UIP controls. DEGs were identified by comparing the expression values in lung tissues between RA-UIP and non-UIP controls via the limma R package in Bioconductor (http://www.bioconductor.org/packages/release/bioc/html/limma.html). The screening criteria were set to adjusted values of *P* < 0.05 and FC > 1.

### Cell culture and siRNA transfection

The human promyelocytic leukemia (HL-60) and fetal lung fibroblast (MRC-5) cell lines were purchased from Pricella Bioscience Inc. (Wuhan, China) and cultured in Iscove’s modified Dulbecco’s medium (IMDM) (Gibco, USA) supplemented with 20% fetal bovine serum (FBS) (Gibco, USA) and 1% antibiotic-antimycotic solution at 37 °C and 5% CO_2_. HL-60 cells were transfected with either si-Control or si-Nr4a3 (Sangon, Shanghai, China) via Lipofectamine 3000 (L3000015, Invitrogen) according to the manufacturer’s instructions. HL-60 cells were incubated with 1 µmol/L all-trans retinoic acid (ATRA R2625-50MG, Sigma) for 4 days to induce neutrophil-like differentiation. Subsequently, the cells were treated with 100 nmol/L PMA and 5% serum from patients with RA-ILD (RA-ILD serum) for 4 h to induce NETosis and the release of NETs.

### Human subjects

The study and protocol were approved by the Ethics Committee for Conducting Human Research at the General Hospital of Ningxia Medical University (KYLL-2024-1033). All patient participants provided written consent for the collection and analysis of their blood samples for publication, in accordance with the protocol (KYLL-2024-1033) outlined by the Ethics Committee. The investigator maintains human research records, including signed and dated consent documents, for 10 years. All cases fulfilling the 2010 American College of Rheumatology (ACR)/European League Against Rheumatism (EULAR) and/or 1987 ACR revised criteria were collected from the outpatient rheumatology and respiratory clinic of the General Hospital of Ningxia Medical University between November 2022 and July 2024 [26]. Pulmonary involvement was assessed in all patients via high-resolution computed tomography (HRCT). The assessments and subsequent reclassification of HRCT images according to the 2013 IIP classification [27] were independently reviewed by two senior radiologists at the General Hospital of Ningxia Medical University, with 8 and 15 years of experience each. Ultimately, 25 RA-nonspecific interstitial pneumonia (NSIP), 24 RA-usual interstitial pneumonia (UIP), 12 RA-organizing pneumonia (OP), and 12 RA-other patterns patients were included in this study (Supplementary Figure S1a). Clinical characteristics and laboratory data were extracted from medical records. The demographics of the individuals involved in this study are outlined in Supplementary Table S3.

### Isolation of human neutrophils from peripheral blood

Peripheral blood (10 mL) was collected from healthy volunteers, RA-UIP patients, and RA-NSIP patients using anticoagulant citrate dextrose. Human neutrophils were isolated from anticoagulated blood via immunomagnetic negative selection using the EasySep™ Direct Human Neutrophil Isolation Kit (Stemcell Technologies, Canada) following the manufacturer’s instructions. The purified neutrophils were used for subsequent experimental procedures. To reproduce ex vivo observations, human neutrophils were stimulated with 100 nmol/L PMA for 2 h to induce NETosis and the release of NETs.

### Statistics

Except for the scRNA-seq data, the experimental data were analyzed via two-tailed unpaired Student’s t tests, one-way or two-way ANOVA with GraphPad Prism software (version 9.5, GraphPad Software, Inc., San Diego, CA) and the “ggplot2”, “ggtext”, “stats”, and “car” packages in R (version 4.2.1). The data are expressed as the means ± standard errors, and differences were considered statistically significant when *P* < 0.05. All experiments, except for the scRNA-seq, were performed independently at least three times.

## Results

### Development of joint swelling and interstitial pneumonia in SKG mice

**Fig. 1:**
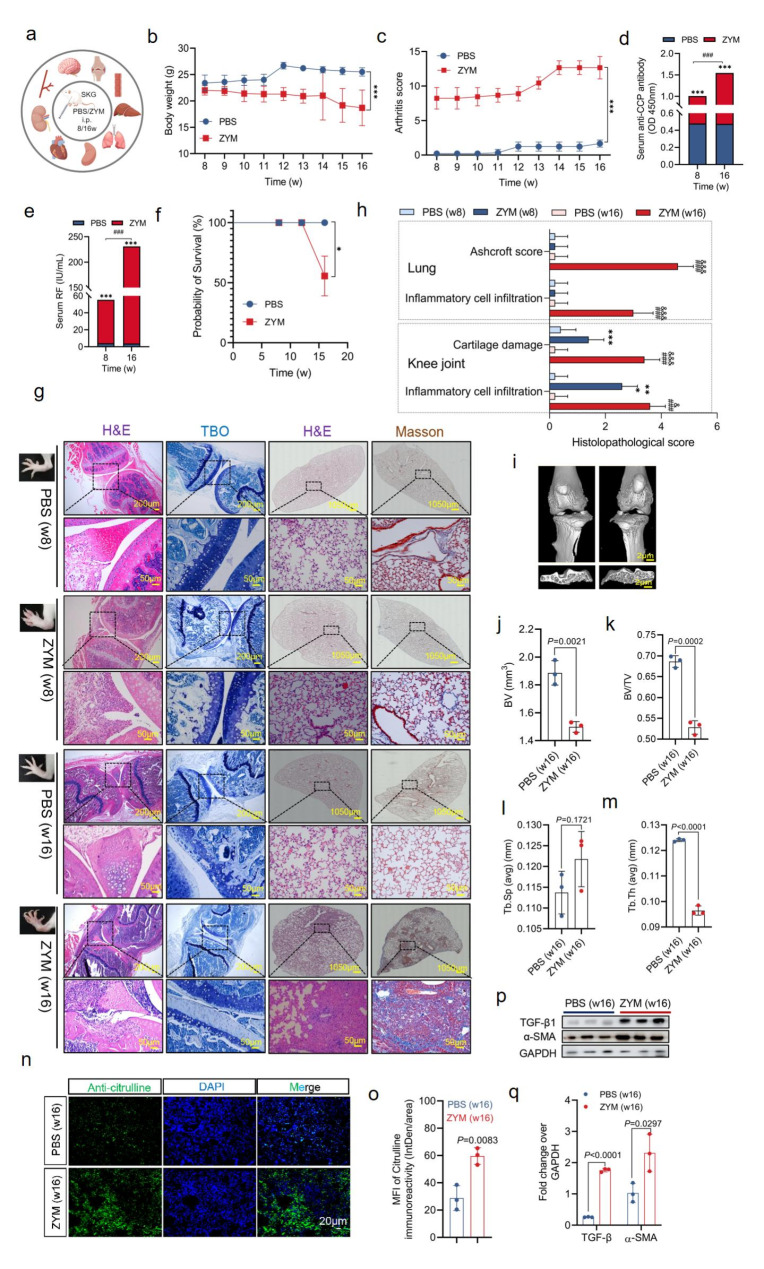
Joint swelling and interstitial pneumonia in SKG mice. **(a)** SKG mice were treated intraperitoneally with 7.5 mg/kg ZYM or PBS to induce joint swelling and interstitial pneumonia. Major organs, including brain, heart, liver, spleen, lung, kidney, colon, knee joint and blood, were harvested at 8 and 16 weeks post-administration. Some figure elements were created with FigDraw.com. **(b, c)** The weight (**b**, *n* = 9,9,9,9,9,9,9,9,6 in each time point ) and arthritis score (**c**, *n* = 9,9,9,9,9,9,9,9,6 in each time point) of SKG mice treated with ZYM and PBS were monitored weekly. **(d, e)** Anti-CCP antibody and RF levels were measured in sera of mice at 8 or 16 weeks post-ZYM or PBS challenge (*n* = 6 per group). **(f)** Overall survival rates of SKG mice treated with ZYM or PBS for 16 weeks. **(g)** Representative external appearance, H&E, TBO, and Masson staining images of knee joints and lungs at 8 and 16 weeks after ZYM stimulation. Scale bars: 200 μm, 1050 μm, 50 μm. **(h)** Tissue sections were graded for cartilage damage, inflammatory cell infiltration, and Ashcroft score. **(i)** µCT images showing knee joints and sagittal views of subchondral bone at 16 weeks post-ZYM or PBS administration. Scale bars = 2 μm. **(j, k, l, m)** Quantitative analysis of BV, BV/TV, Tb.Sp and Tb.Th. **(n, o)** Immunofluorescence and statistical analysis of anti-citrullinated protein in the lungs of ZYM and PBS-treated mice (*n* = 3 in each group). Nuclei were stained with DAPI (blue). Scale bars = 20 μm. **(p, q)** Immunoblots and statistical analysis of TGF-β and α-SMA expression in the lungs ( *n* = 3 in each group). ZYM: zymosan A; anti-CCP: anti-cyclic citrullinated peptide; RF: rheumatoid factor; BV: bone volume; BV/TV: bone volume/total tissue volume; Tb.Sp: trabecular separation; Tb.Th: trabecular thickness; TGF-β: transforming growth factor beta; α-SMA: alpha-smooth muscle actin. *P < 0.05; **P < 0.01; ***P < 0.001; ****P < 0.0001 vs. PBS (w8); ^#^*P* < 0.05; ^##^*P* < 0.01; ^###^*P* < 0.001; ^####^*P* < 0.0001 vs. ZYM (w8)

To investigate the progression of lung disease in the context of arthritis, we induced chronic arthritis and interstitial pneumonia in SKG mice maintained under SPF conditions. ZYM was administered intraperitoneally at 8 and 16 weeks of age (Fig. 1a). Compared with the PBS-treated control mice, the ZYM-treated mice had significantly impaired growth (*P* < 0.0001) (Fig. 1b). The arthritis scores of the ZYM-treated mice were markedly greater than those of the PBS-treated controls (*P* < 0.0001) (Fig. 1c). Key diagnostic indicators, including circulating anti-CCP (Fig. 1d) and RF (Fig. 1e), were significantly elevated in ZYM-treated mice than in control mice at both 8 and 16 weeks post challenge (*P* < 0.0001 and *P* < 0.0001, respectively). Additionally, whereas the mortality rate of the PBS-treated mice was 0% during the 16-week observation period, only 50% of the ZYM-treated mice survived to the end of the study (*P* = 0.0275) (Fig. 1f). Histological analysis revealed substantial inflammatory cell infiltration in the knee joints, colon, and spleen of the mice at 8 weeks post-ZYM challenge (Fig. 1g and S1b). Notably, SKG mice treated with ZYM for 16 weeks presented systemic manifestations akin to those of human RA, including joint swelling and deformity, colonic inflammation, splenomegaly, hepatic inflammation, and interstitial pneumonia (Fig. 1g and S1b). TBO analysis revealed pronounced destruction of the articular cartilage, whereas Masson’s trichrome staining revealed significant collagen deposition around the vascular walls and in the thickened alveolar interstitium at 16 weeks post-ZYM challenge (Fig. 1g). Histopathological analysis revealed a significant increase in knee joint inflammatory scores in ZYM-treated mice at week 8 (2.6 ± 0.54) compared with those in PBS-treated controls (0.2 ± 0.44) (*P* < 0.0001) (Fig. 1h). At week 16 post-ZYM treatment, the mice presented increased inflammatory scores (3.6 ± 0.54) and articular cartilage scores (3.4 ± 0.54) in the knee joint, as well as elevated inflammatory (3.0 ± 0.71) and fibrotic scores (2.4 ± 0.55) in the lung compared with those of the controls (*P* < 0.0001 for all) (Fig. 1g). Micro-CT revealed significant bone resorption induced by ZYM, as evidenced by increased osteolysis in the subchondral bone of the tibia (Fig. 1i). Quantitative analysis revealed that the BV of the subchondral bone in the tibial plateau was significantly reduced in the ZYM-treated group (1.49 ± 0.04 mm^3^) compared to the PBS control group (1.88 ± 0.08 mm^3^) (Fig. 1j). Additional parameters, such as BV/TV, Tb.N, and Tb.Sp, also indicated bone destruction in the ZYM-treated group (Fig. 1k and l, and 1m). Furthermore, IF analysis revealed increased levels of citrullinated peptides in the lungs of ZYM-treated mice at 16 weeks (Fig. 1n and o). Immunoblotting further revealed elevated expression of TGF-β1 and the profibrogenic factor alpha smooth muscle actin (α-SMA) in the lungs of ZYM-treated mice compared with those in the lungs of control mice at 16 weeks (Fig. 1p and q). These results confirmed that SKG mice treated with ZYM for 16 weeks developed significant ILD under SPF conditions.

### Single-cell transcriptomic profiling increased the number of neutrophils in SKG mice with joint swelling and interstitial pneumonia

**Fig. 2:**
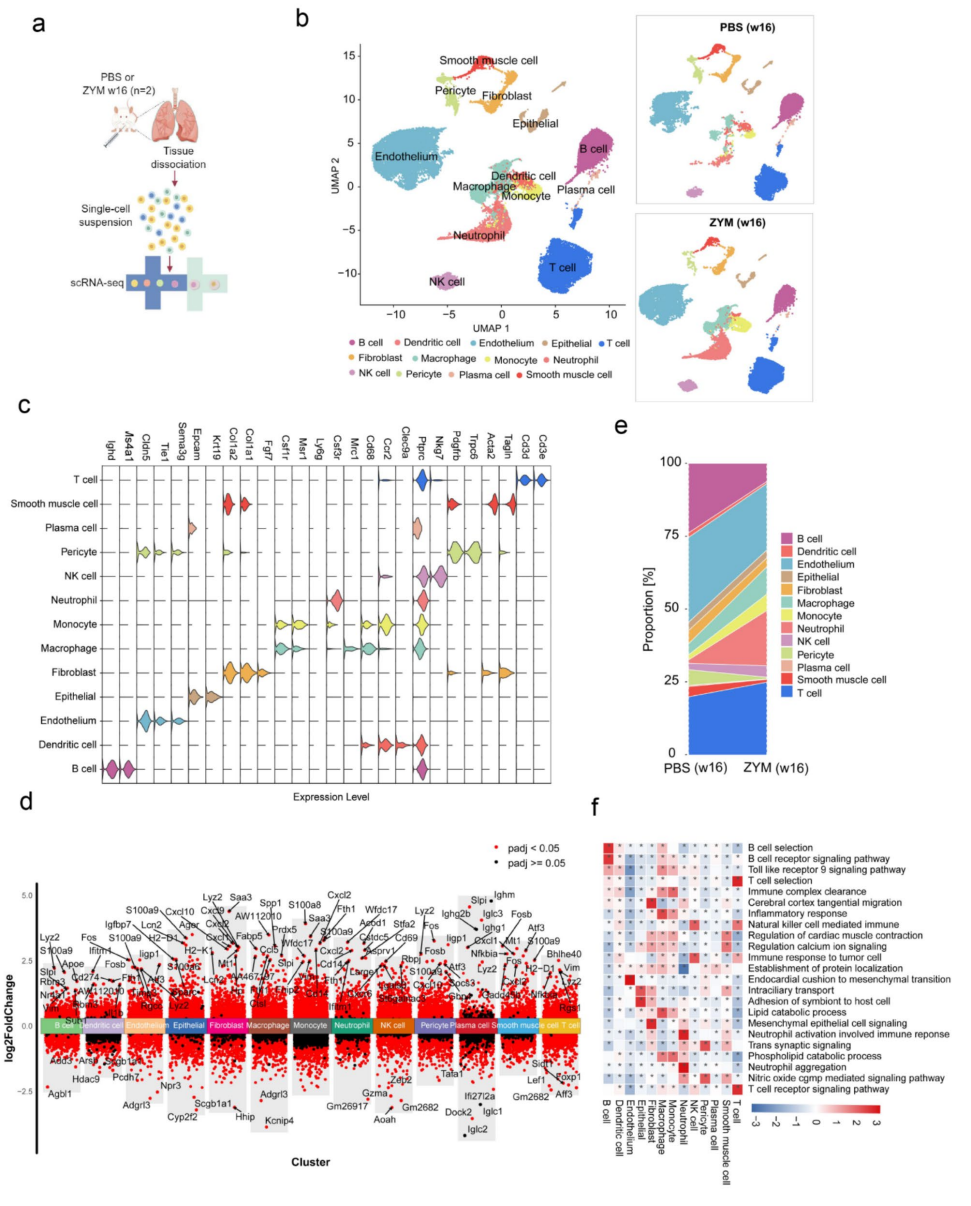
Single-cell transcriptomic profiling increased neutrophils in SKG mice with joint swelling and interstitial pneumonia. **(a)** Graphical representation of the experimental workflow for single-cell sequencing study. Some figure elements were created with FigDraw.com. **(b)** UMAP plot showing the nine cell types in lungs of mice at w16 post-PBS (17,770 cells) or ZYM (19,965 cells) challenge. Each point depicts a single cell, colored based on the cluster designation. **(c)** Violin plots showing the expression levels of representative cell type-specific marker genes in the mouse lung. **(d)** Upregulated and downregulated genes across the thirteen clusters according to differential gene expression analysis. **(e)** Bar graph showing variations in the proportion of cell subsets among the PBS control and ZYM stimulation group. **(f)** GO analysis showing the specific functional characteristics of each cell type. FC indicates fold change; the red dot indicates the adjusted *P* < 0.05; the green dot indicates adjusted *P* > 0.05. UMAP: Uniform manifold approximation and projection; GO: Gene Ontology

To obtain a high-resolution map of the mouse lung under both normal and pathological conditions, we utilized single-cell RNA sequencing (scRNA-seq). Lung tissues were harvested from SKG mice 16 weeks after treatment with either ZYM or PBS, followed by rapid digestion into single-cell suspensions. The samples were then processed via a single-tube protocol for unique transcript counting by barcoding with unique molecular identifiers (UMIs) on the 10× Genomics Chromium platform (Fig. 2a). Following quality control filtering, a total of 37,735 cells were analyzed, with an average of 5,789 genes detected per cell. Among these cells, 17,770 originated from PBS-treated SKG mice, whereas 19,965 were obtained from ZYM-treated mice. The uniform manifold approximation and projection (UMAP) algorithm was employed to visualize the cells, and the cell types were annotated based on previously reported markers [28]. We identified 11 major cell types within the lung, including B lymphocytes (B cells), dendritic cells, endothelial cells, epithelial cells, fibroblasts, macrophages, monocytes, neutrophils, natural killer (NK) cells, pericytes, plasma cells, smooth muscle cells, and T lymphocytes (T cells) (Fig. 2b). Marker genes specific to each cell type were also identified (Fig. 2c). Furthermore, differential expression analysis was conducted to determine the most significant DEGs in each cluster between ZYM- and PBS-treated mice, enabling manual annotation of each cluster’s cellular identity (Fig. 2d). We next compared the relative cell populations in the lung samples of mice treated with PBS and ZYM. As illustrated in Fig. 2e, ZYM treatment resulted in a marked increase in neutrophil numbers while concomitantly reducing the populations of B cells and pericytes. To elucidate the physiological functions of these cell populations, we performed GO enrichment analysis based on the expression profiles of the top cell type-specific genes. Notably, neutrophils were significantly enriched in biological processes such as neutrophil aggregation and neutrophil activation, which are involved in the immune response (Fig. 2f). Collectively, these data demonstrated a pronounced expansion of the neutrophil population within the lung tissue of ZYM-treated mice.

### Increased neutrophil extracellular traps (NETs) in SKG mice with joint swelling and interstitial pneumonia

**Fig. 3:**
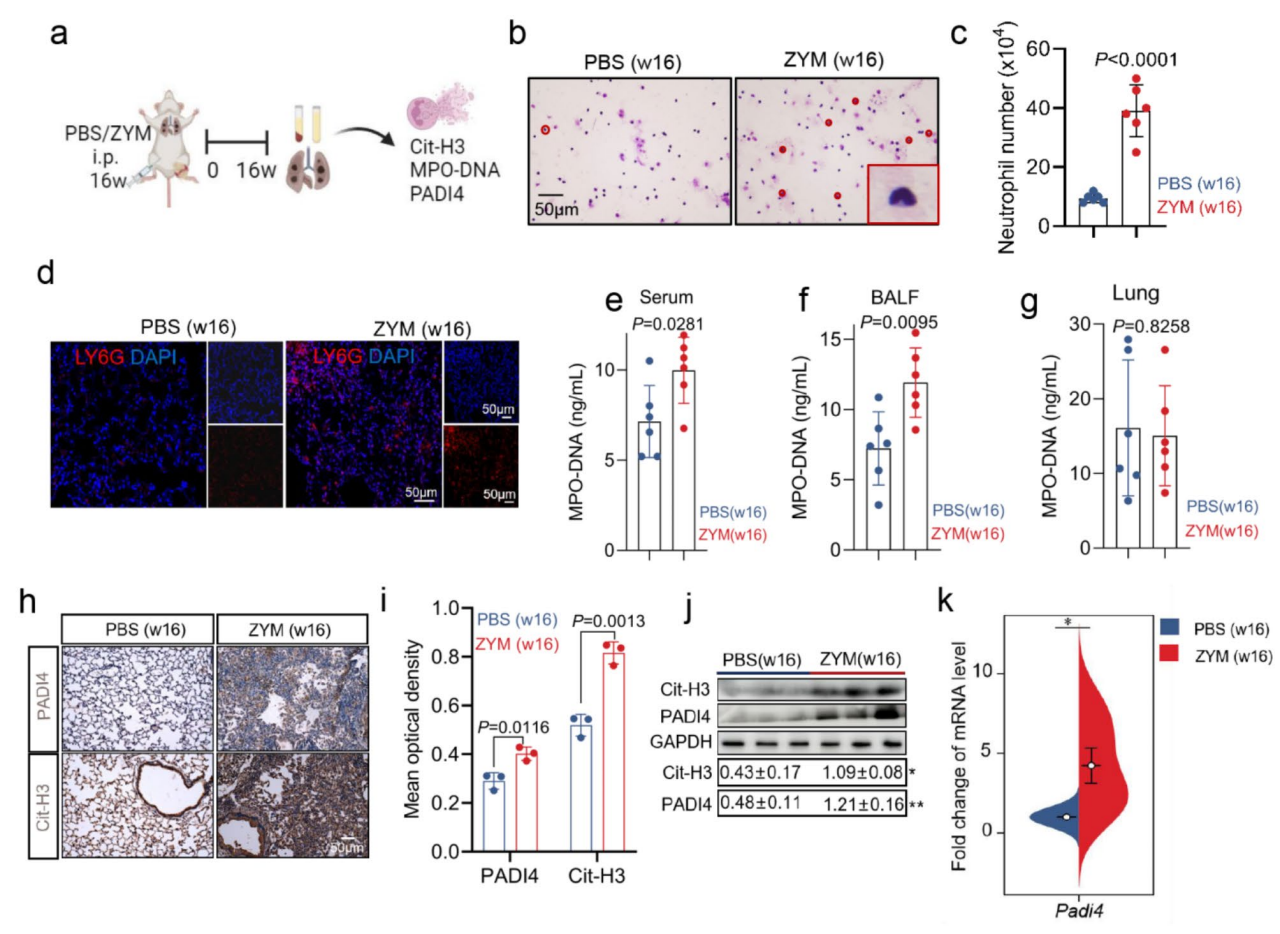
Increased Neutrophil Extracellular Traps in SKG mice with joint swelling and interstitial pneumonia. **(a)** Flowchart of sample collection and analysis. **(b, c)** Animals were euthanized and BALF was evaluated for neutrophils (**c**, n = 6 in each group), as well as Giemsa staining **(b)** at 16 w after ZYM and PBS challenge. In panel **(b)**, red dots indicate PMNs, with a magnified view of the PMNs shown in the lower right panel. Scale bars = 50µm. **(d)** Immunofluorescent staining the expression of neutrophil marker Ly6g in the lungs of mice treated with ZYM and PBS. Nuclei were stained with DAPI (4’,6-Diamidino-2-phenylindole), displayed in blue. Scale bars = 50 μm. **(e, f, g)** Levels of MPO-DNA in serum (**e**, *n* = 6 in each group), BALF (**f**, *n* = 6 in each group) and lung tissue (**g**, *n* = 6 in each group) at 16w after ZYM and PBS stimulation. **(h, i)** Immunohistochemical staining and quantitative analysis of Cit-H3 and PADI4 (*n* = 3 in each group) in mouse lung samples. **(j)** Immunoblots and statistical analysis showing the expression of PADI4 and Cit-H3 in the lungs of mice treated with ZYM and PBS (*n* = 3 in each group). **(k)** The mRNA level of *padi4* (*n* = 6 in each group) in lung samples from SKG mice treated with ZYM and PBS for a period of 16 weeks. PMNs: polymorphonuclear neutrophils; MPO-DNA: myeloperoxidase-DNA; Cit-H3: citrullinated histone H3; padi4: peptidyl arginine deiminase 4. *P < 0.05; **P < 0.01; ***P < 0.001; ****P < 0.0001 as compared with the PBS (w16)

The aforementioned data suggest that ZYM-induced neutrophils primarily contribute to functions related to NET formation. To further investigate this hypothesis, we collected bronchoalveolar lavage fluid (BALF), blood, and lung tissue 16 weeks after ZYM exposure to assess neutrophil counts and NET levels (Fig. 3a). As shown in Fig. 3b-d, ZYM treatment significantly increased neutrophil infiltration in the BALF (*P* < 0.0001) (Fig. 3b and c), accompanied by a notable increase in the neutrophil marker Ly6g in the lung tissue (*P* < 0.0001) (Fig. 3d). ELISAs revealed elevated levels of MPO-DNA, a key NET component, in both the serum and BALF at 16 weeks post-ZYM exposure compared with those in the PBS treatment (*P* = 0.0281 and *P* = 0.0095, respectively) (Fig. 3e and f). Interestingly, no significant difference in the lung MPO-DNA concentration was detected between ZYM-treated and PBS-treated SKG mice (Fig. 3g). Molecular analysis via immunohistochemistry and immunoblotting revealed increased protein levels of Cit-H3 and PADI4 (Fig. 3h, i, and j) and increased transcript levels of *padi4* in the lungs of ZYM-treated mice compared with those in the PBS group (Fig. 3k). These data suggest that NETs may contribute to the pathogenesis of interstitial pneumonia during the progression of joint swelling in SKG mice.

### NR4A3 inhibits NET formation induced by RA-ILD patient serum

**Fig. 4:**
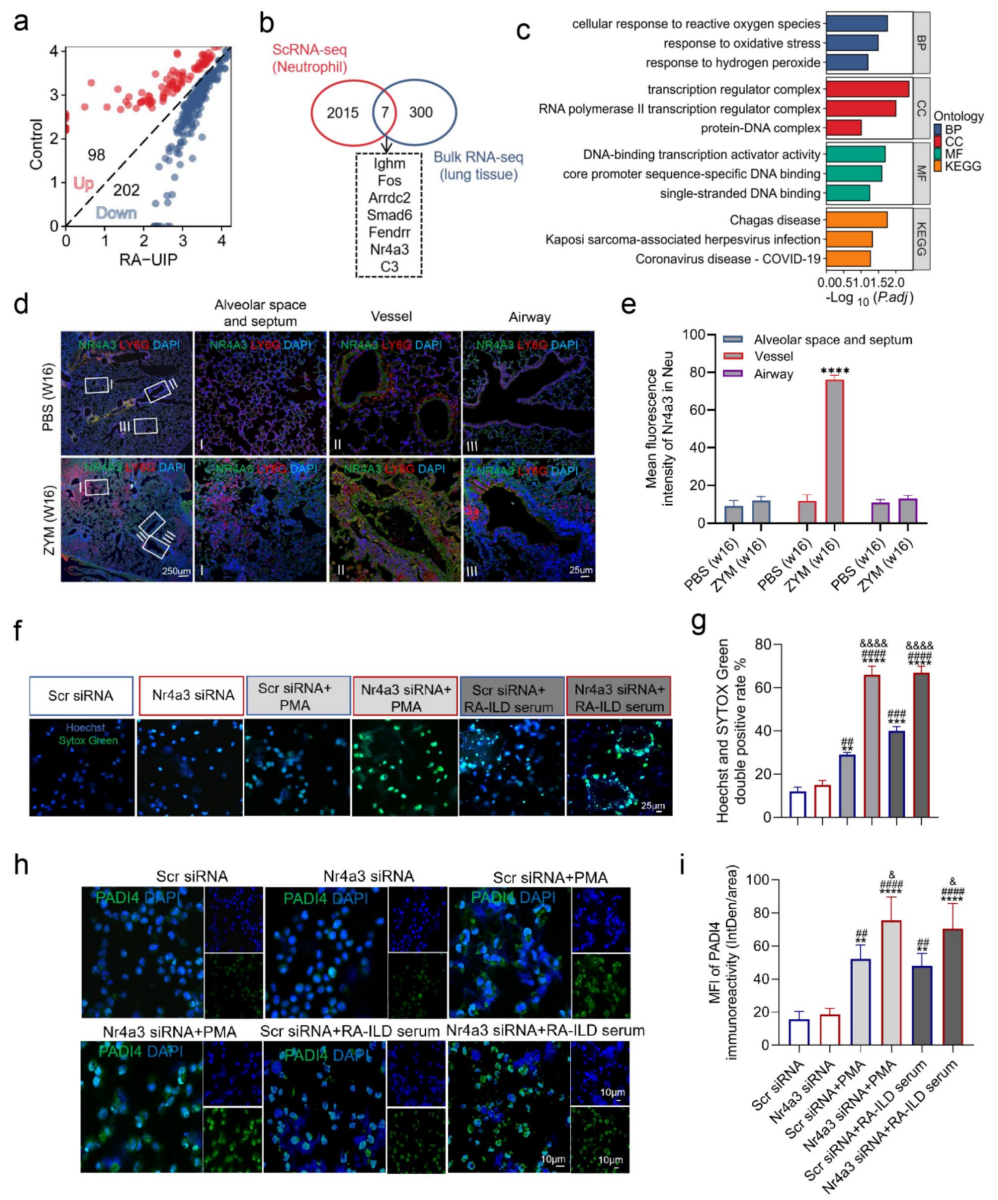
NR4A3 inhibits NET formation induced by RA-ILD patient serum. **(a)** Diagonal volcano plot analysis of DEGs from dataset GSE199152 was conducted using a cutoff criterion of P < 0.05 and log2 fold change > 1. A total of 300 DEGs were identified in GSE199152. **(b)** Venn diagram analysis identified seven co-DEGs from the 2,015 DEGs of GSE199152 and 300 DEGs from lung neutrophils. **(c)** GO analysis shows the specific functional characteristics of seven co-DEGs. **(d)** Immunofluorescence staining with Nr4a3/Ly6g was used to detect the distribution in the lungs of mice treated with ZYM and PBS. Nuclei were stained with DAPI (4’,6-diamidino-2-phenylindole), displayed in blue. Scale bars: 250 μm in the left columns; 25 μm in the right three columns. **(e)** Quantitative analysis of the mean fluorescence intensity of Nr4a3 in the lungs of mice treated with ZYM and PBS. **(f, g)** Investigation into the effect of NR4A3 on NETs secretion induced by PMA and RA-ILD serum in HL-60 cells. Cells in 96-well plates were treated with Scr siRNA, Nr4a3 siRNA, Scr siRNA + PMA, Nr4a3 siRNA + PMA, Scr siRNA + RA-ILD serum, or Nr4a3 siRNA + RA-ILD serum, and stained for total DNA (blue) and cfDNA (green). Hoechst and SYTOX Green double-positive cells were defined as undergoing NETosis. Scale bar: 25 μm. **(h, i)** Analysis of PADI4 in HL-60 cells treated under various conditions using IF (*n* = 3). Scale bar: 10 μm. Co-DEGs: common differentially expressed genes; GSE: gene expression series; GO: Gene Ontology; Scr: scramble. *P < 0.05; **P < 0.01; ***P < 0.001; ****P < 0.0001 as compared with the PBS (w16) and Scr siRNA groups, respectively; ^#^*P* < 0.05; ^##^*P* < 0.01; ^###^*P* < 0.001; ^*####*^*P* < 0.0001 as compared with the Nr4a3 siRNA group; ^&^*P* < 0.05; ^&&^*P* < 0.01; ^&&&^*P* < 0.001; ^&&&&^*P* < 0.0001 as compared with the Scr siRNA + PMA group

RA-UIP is one of the most common forms of ILD and has a poor prognosis [28]. We analyzed data from the NCBI GEO profile (GSE199152) to identify DEGs in the lung tissues of RA-UIP patients compared with non-UIP controls. A total of 300 DEGs were identified, consisting of 98 upregulated genes and 202 downregulated genes (Fig. 4a). By comparing DEGs from our scRNA-seq analysis of neutrophils with those from bulk RNA-seq of lung tissues, we identified seven common DEGs, including immunoglobulin heavy constant mu (IGHM), fos proto-oncogene (FOS), alpha arrestin domain containing 2 (ARRDC2), SMAD family member 6 (SMAD6), FOXF1 adjacent noncoding developmental regulatory RNA (FENDRR), nuclear receptor subfamily 4 group A member 3 (NR4A3), and complement C3 (C3) (Fig. 4b). GO analysis indicated that these seven common DEGs were involved primarily in the cellular response to reactive oxygen species and oxidative stress (Fig. 4c), suggesting a potential role in NET formation. IF further revealed increased numbers of NR4A3 and LY6G double-positive neutrophils in the blood vessels but not in the bronchi, alveolar space, or septum (Fig. 4d and e). To investigate the role of NR4A3 in NET formation, we inhibited Nr4a3 expression via siRNA (si-Nr4a3) in HL-60 cells. The first Nr4a3 siRNA efficiently silenced the endogenous NR4A3 protein in HL-60 cells and was therefore used in this study (Figure S2a). PMA stimulation, as a positive control for NET formation, increased Hoechst and SYTOX Green double-positive cells and PADI4 expression in HL-60 cells. The serum of RA-ILD patients also increased the levels of these markers after 2 h of stimulation. Notably, the inhibition of NR4A3 resulted in a further increase in the number of Hoechst and SYTOX Green double-positive cells and PADI4 expression induced by RA-ILD serum after 2 h (Fig. 4f, g, h, and i). Collectively, these data suggest that NR4A3 inhibits the formation of NETs.

### NETs from Nr4a3-depleted HL-60 cells induce the differentiation of MRC-5 cells into myofibroblasts

**Fig. 5:**
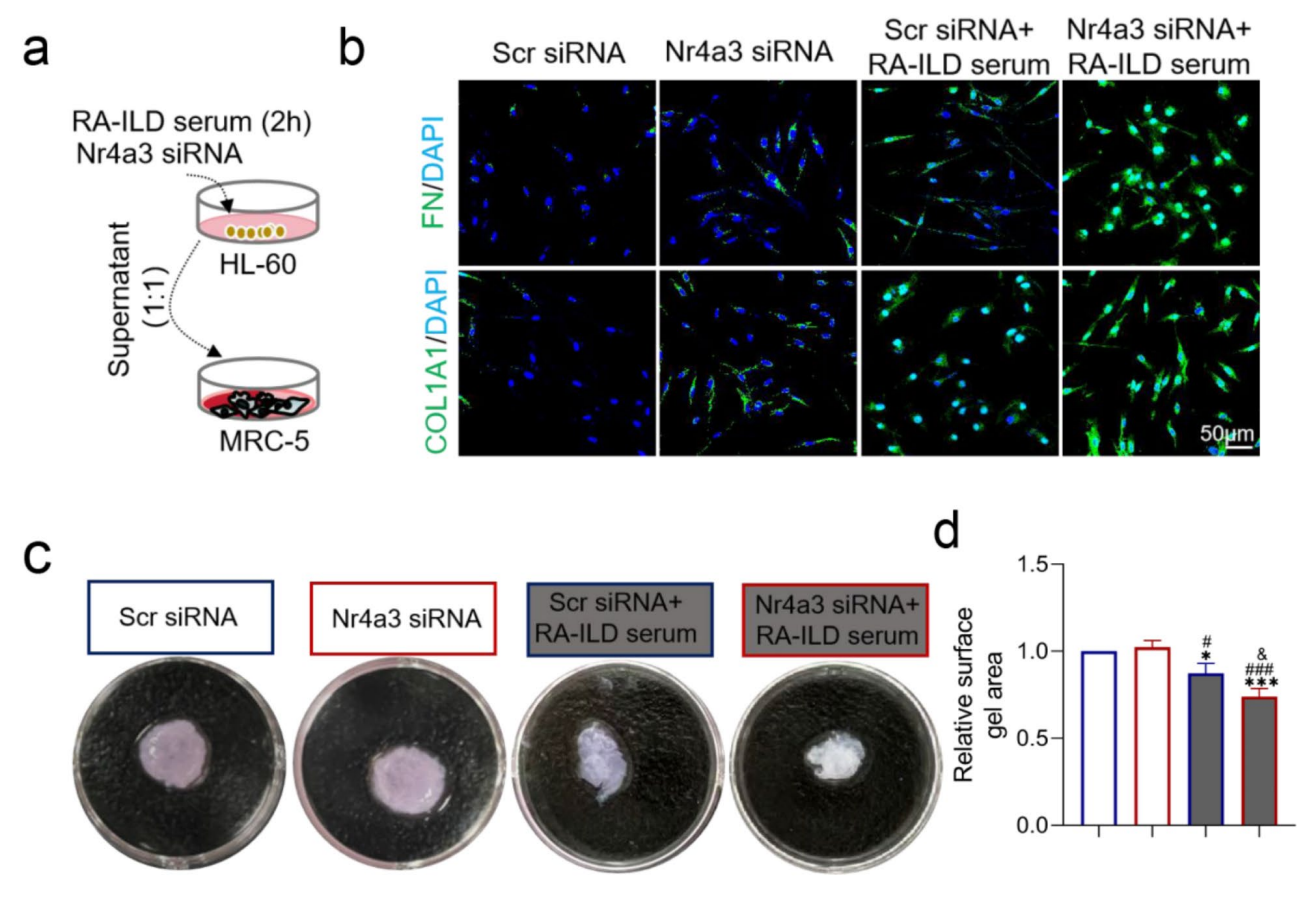
NETs from Nr4a3-depleted HL-60 cells induce the differentiation of MRC-5 cells into myofibroblasts. **(a)** A flow chart illustrating the co-culture model of HL-60 and MRC-5 cells. **(b)** Expression of myofibroblast markers FN and COL1A1 in MRC-5 cells was analyzed after exposure to supernatants from HL-60 cells for 48 h through IF. **(c, d)** Representative images and quantification of gel contraction assays demonstrate the functional impact of HL-60 supernatants on MRC-5 cells for 48 h of exposure. FN: fibronectin; COL1A1: collagen type 1; PMA: phorbol 12-myristate 13-acetate. *P < 0.05; **P < 0.01; ***P < 0.001; ****P < 0.0001 as compared with the Scr siRNA; ^#^*P* < 0.05; ^##^*P* < 0.01; ^###^*P* < 0.001; ^####^*P* < 0.0001 as compared with the Nr4a3 siRNA; ^&^*P* < 0.05; ^&&^*P* < 0.01; ^&&&^*P* < 0.001; ^&&&&^*P* < 0.0001 as compared with the Scr siRNA + PMA

Activated fibroblasts are central to the progression of pulmonary fibrosis [29]. To investigate the effect of NETs on MRC-5 differentiation, supernatants were collected from HL-60 cells transfected with either Scr siRNA or Nr4a3 siRNA in the presence or absence of RA-ILD patient serum. The supernatants were then mixed with MRC-5 cell culture medium at a 1:1 ratio. After 48 h of exposure, we assessed the expression of the myofibroblast markers fibronectin (FN) and collagen 1 (COL1A1) in MRC-5 cells (Fig. 5a). IF staining revealed that supernatants from RA-ILD patient serum-treated HL-60 cells transfected with Nr4a3 siRNA significantly increased the abundance of FN and COL1A1 in MRC-5 cells compared with supernatants from HL-60 cells treated with RA-ILD serum alone (Fig. 5b). Collagen gel contraction assays further confirmed enhanced collagen gel contraction in the supernatants from Nr4a3-depleted HL-60 cells treated with RA-ILD patient serum compared with those from HL-60 cells treated with RA-ILD serum alone (*P* < 0.0001) (Fig. 5c and d). These data suggest that NR4A3-mediated suppression of NET secretion inhibits the differentiation of MRC-5 cells into myofibroblasts.

### Increased circulating MPO-DNA in RA-NSIP patients and Cit-H3 in RA-UIP patients

**Fig. 6:**
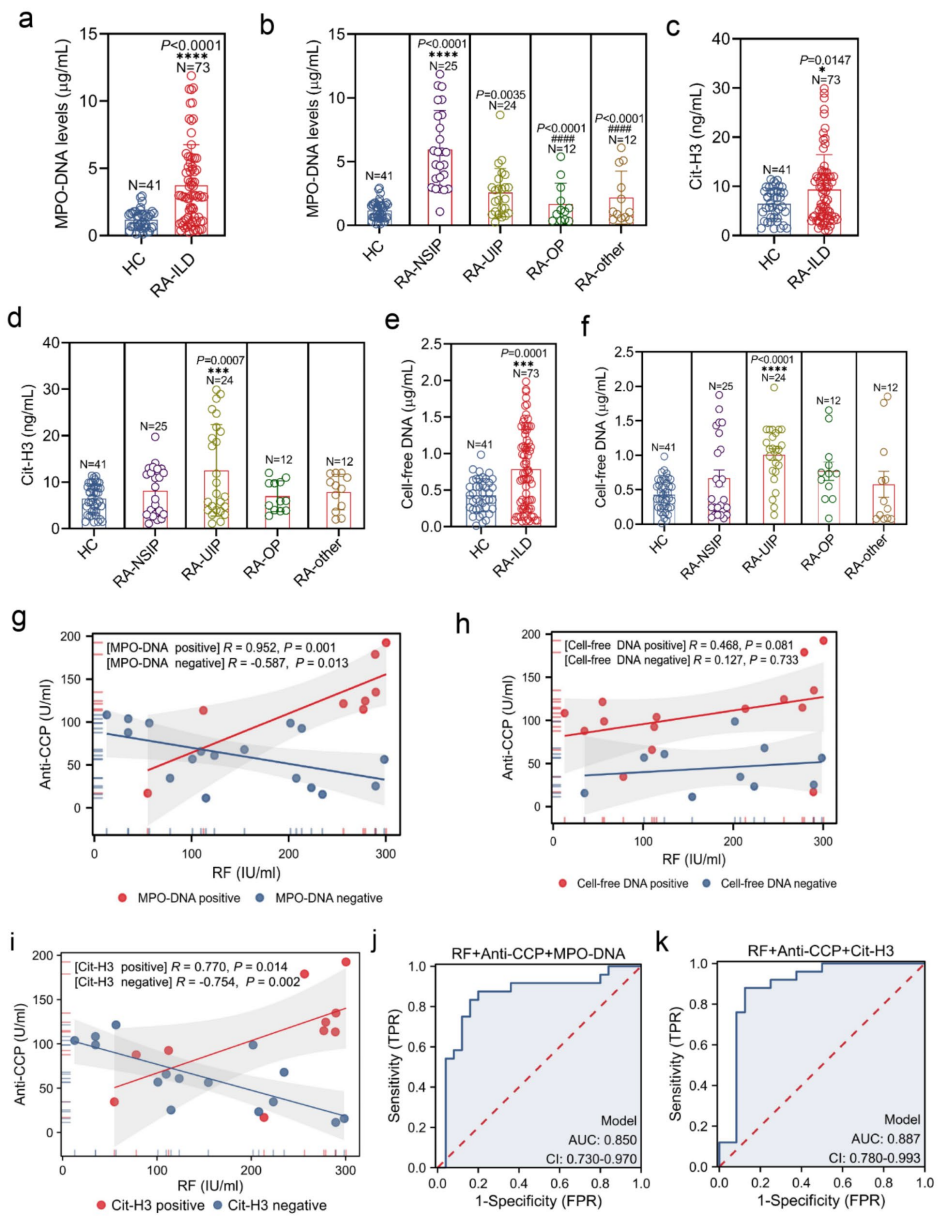
Increased circulation MPO-DNA in RA-NSIP patients and Cit-H3 in RA-UIP patients. **(a, c, e)** Plasma concentrations of MPO-DNA **(a)**, Cit-H3 **(c)**, and cell-free DNA **(e)** were measured in healthy individuals and RA-ILD patients. **(b, d, f)** Plasma concentrations of MPO-DNA **(b)**, Cit-H3 **(d)**, and cell-free DNA **(f)** were measured in HC cohorts (*n* = 41), RA-UIP (*n* = 25), RA-NSIP (*n* = 24), RA-OP (*n* = 12), and RA-ILD patients with other patterns (*n* = 12). **(g, h, i)** Correlations between anti-CCP antibody and RF in RA-NSIP patients with MPO-DNA positivity (*n* = 8) and negativity (*n* = 17) **(g)**, RA-UIP patients with Cit-H3 positivity (*n* = 10) and negativity (*n* = 15) **(h)**, and RA-UIP patients with cell-free DNA positivity (*n* = 15) and negativity (*n* = 10) **(i)** were detected by Spearman test. **(j)** A ROC curve demonstrating the diagnostic potential of integrating plasma MPO-DNA, RF, and anti-CCP antibody for RA-NSIP. **(k)** A ROC curve showing the diagnostic potential of integrating plasma Cit-H3, RF, and anti-CCP antibody for RA-UIP. *P < 0.05; **P < 0.01; ***P < 0.001; ****P < 0.0001 as compared with the HC cohorts; ^#^*P* < 0.05, ^##^*P* < 0.01; ^###^*P* < 0.001; ^####^*P* < 0.0001 as compared with RA-UIP patients. MPO-DNA: myeloperoxidase-DNA; Cit-H3: citrullinated histone H3; HRCT: high-resolution computed tomography; ILD: interstitial lung disease; RA: rheumatoid arthritis; UIP: usual interstitial pneumonia; NSIP: nonspecific interstitial pneumonia; OP: organizing pneumonia

Unlike SKG mice, which exhibit joint swelling and interstitial pneumonia, plasma neutrophil counts did not significantly differ between RA-ILD patients with varying imaging features and healthy individuals (Figure S2b). To determine whether NETs are correlated with RA-ILD activity, the plasma concentrations of MPO-DNA, Cit-H3, and cell-free DNA were evaluated in RA-ILD patients with different imaging features and healthy individuals. Compared with those in healthy individuals, plasma MPO-DNA levels were significantly greater in RA-ILD patients (*P* < 0.00001) (Fig. 6a). Similarly, compared with healthy individuals, RA-NSIP patients also presented markedly elevated levels of plasma MPO-DNA (*P* < 0.00001) (Fig. 6b). Similarly, the plasma concentrations of Cit-H3 and cell-free DNA were also significantly greater in RA-ILD patients than in healthy controls (*P* = 0.0147 and *P* < 0.0001, respectively) (Fig. 6c and e). However, RA-UIP patients had moderately higher levels of plasma Cit-H3 and cell-free DNA than healthy subjects (*P* = 0.0007 and *P* < 0.00001, respectively) (Fig. 6d and f). We next analyzed the correlations between RA-ILD serological risk features, including RF and anti-CCP, and plasma concentrations of MPO-DNA, Cit-H3 and cell-free DNA. In the MPO-DNA-positive RA-NSIP group, RF was positively correlated with anti-CCP (*R* = 0.952, *P* = 0.001), whereas a negative correlation was found in the MPO-DNA-negative RA-NSIP group (*R*=-0.587, *P* = 0.013) (Fig. 6g). A strong positive correlation between RF and anti-CCP was also detected in the Cit-H3-positive RA-UIP group (*R* = 0.770, *P* = 0.014), whereas a strong negative correlation was detected in the Cit-H3-negative RA-UIP group (*R*=-0.754, *P* = 0.002) (Fig. 6h). Interestingly, no significant correlation was detected between RF and anti-CCP based on the cell-free DNA status of RA-UIP patients (*R* = 0.468, *P* = 0.081 and *R* = 0.127, *P* = 0.733) (Fig. 6i). Finally, we evaluated the clinical utility of MPO-DNA and Cit-H3 as biomarkers for diagnosing RA-ILD with UIP and NSIP features. The area under the curve (AUC) for the combination of circulating MPO-DNA with RF and anti-CCP in RA-NSIP diagnosis was 0.850 (range: 0.730–0.970) (Fig. 6j). Similarly, the inclusion of circulating Cit-H3 with RF and anti-CCP yielded an AUC of 0.887 (range: 0.780–0.993) for RA-UIP diagnosis (Fig. 6k). These results strongly suggest a link between circulating MPO-DNA and RA-NSIP activity, as well as between Cit-H3 and RA-UIP activity.

### Neutrophils from RA-UIP are more prone to NET formation and induce the differentiation of MRC-5 cells into myofibroblasts

**Fig. 7:**
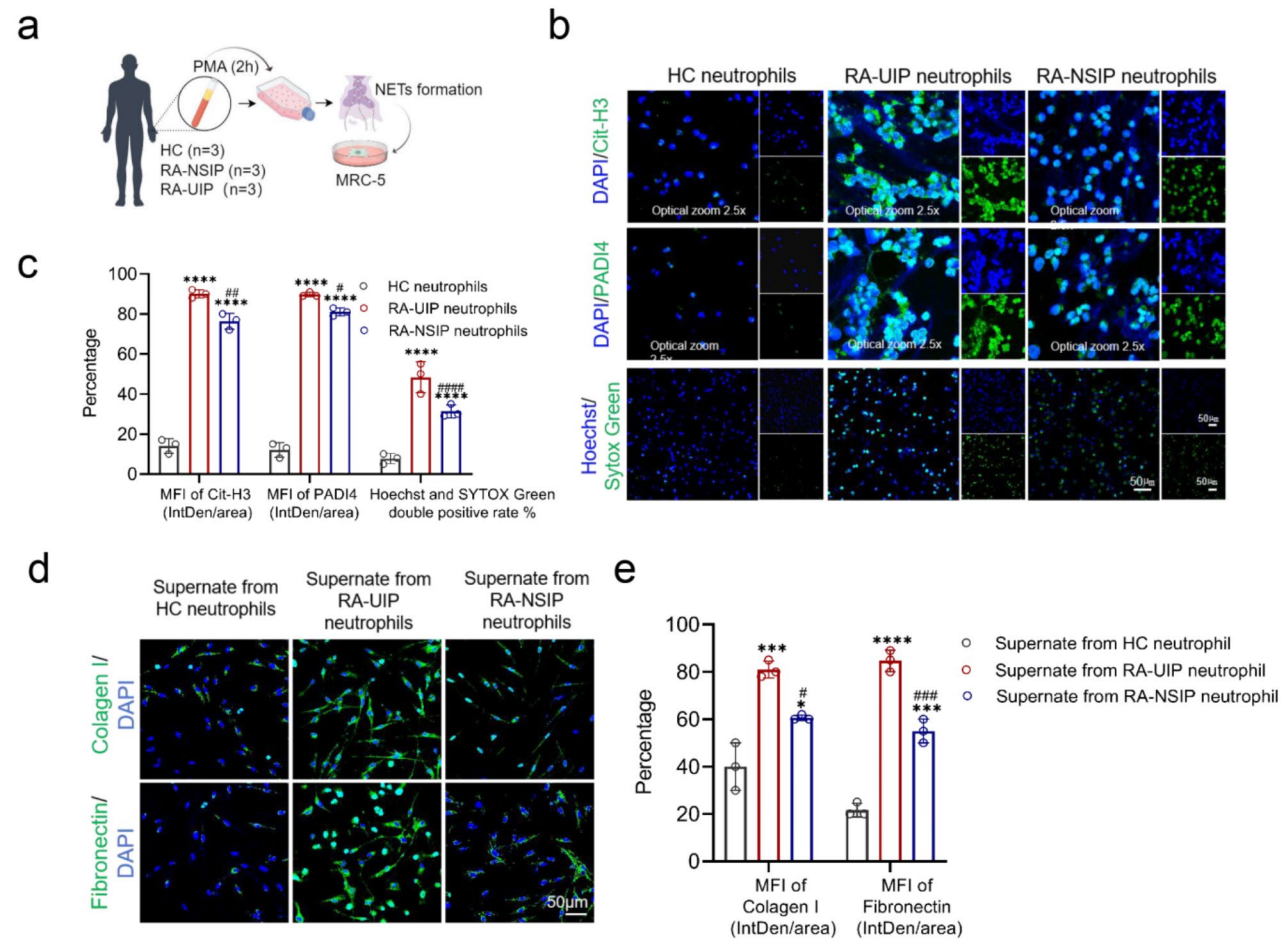
Neutrophils from RA-UIP are more prone to NET formation and induce the differentiation of MRC-5 cells into myofibroblasts. **(a)** A flow chart illustrating the co-culture model of human peripheral neutrophils and MRC-5 cells. **(b, c)** Representative immunofluorescence images and quantification of the human neutrophils treated with PMA for 2 h with staining for Cit-H3 and PADI4. Scale bars, 50 μm. Optical zoom 2.5×. **(d, e)** Representative immunofluorescence images and quantification of FN and COL1A1 in MRC-5 cells after exposure to supernatants from human peripheral neutrophils for 48 h. FN: fibronectin; COL1A1: collagen type 1; PMA: phorbol 12-myristate 13-acetate. Scale bars, 50 μm. *P < 0.05; **P < 0.01; ***P < 0.001; ****P < 0.0001 as compared with the HC group; ^#^*P* < 0.05; ^##^*P* < 0.01; ^###^*P* < 0.001; ^*####*^*P* < 0.0001 as compared with the RA-UIP group

To investigate the associations between RA-ILD imaging patterns and NET formation, neutrophils were isolated from three healthy volunteers, three RA-UIP patients, and three RA-NSIP patients (Fig. 7a). NET formation was assessed in neutrophils 2 h after PMA stimulation. The levels of Cit-H3 and PADI4 were significantly greater in neutrophils from both RA-UIP patients and RA-NSIP patients than in those from healthy controls (Fig. 7b and c). Notably, the levels of Cit-H3 and PADI4 were significantly greater in the neutrophils of RA-UIP patients than in those of RA-NSIP patients (Fig. 7b and c). To further examine the impact of NETs from RA-UIP and RA-NSIP patients on MRC-5 differentiation, supernatants were collected from neutrophils and mixed with MRC-5 cell culture medium at a 1:1 ratio. After 48 h of exposure, IF staining revealed that, compared with those from healthy volunteers, supernatants from both RA-UIP and RA-NSIP patient neutrophils significantly increased the abundance of FN and COL1A1 in MRC-5 cells (Fig. 7d and e). Notably, FN and COL1A1 levels were significantly higher in MRC-5 cells exposed to supernatants from RA-UIP patient neutrophils than in those exposed to supernatants from RA-NSIP patient neutrophils (Fig. 7d and e). These results suggest that neutrophils from RA-UIP patients are more prone to NET formation and induce the differentiation of MRC-5 cells into myofibroblasts than neutrophils from RA-NSIP patients.

## Discussion

The pathogenesis of RA-ILD remains incompletely understood, in part due to the lack of appropriate animal models [30]. Conventional models, such as collagen-induced arthritis (CIA) models, effectively induce inflammatory arthritis but fail to adequately replicate the pulmonary fibrosis that commonly complicates RA [31]. This limitation highlights the need for more suitable experimental models to better explore the pathogenesis of RA-ILD. In the present study, we utilized SKG mice to establish a model of a chronic progressive joint disorder, which was accompanied by the development of cellular and fibrotic interstitial pneumonia. Consistent with previous reports, SKG mice presented increased collagen deposition; however, they did not develop a fibrotic UIP phenotype, even 16 weeks after ZYM injection. The lung pathology observed in SKG mice is similar to that of NSIP observed in humans and is characterized by inflammatory cell infiltration and varying degrees of collagen deposition in areas of cellular accumulation. These findings suggest that the pattern of lung disease in SKG mice closely mirrors the fibrotic NSIP pathology observed in human RA-ILD [31–33]. Furthermore, key serological markers commonly elevated in RA-ILD patients, such as RF and anti-CCP [34], were also elevated in ZYM-treated SKG mice. Additionally, increased levels of anti-CCP, which targets pancitrullinated proteins, along with key markers of fibrotic lung disease, such as TGF-β1 and α-SMA, were detected in the lung tissues of ZYM-injected SKG mice. Our findings, in conjunction with those of previous reports, demonstrate that arthritis SKG mice develop persistent, restrictive mixed cellular and fibrotic interstitial pneumonia that closely resembles the pathogenesis of RA-ILD [19, 30, 32].

Although the mechanism underlying the aberrant regulation of inflammatory cascades and tissue remodeling in various forms of RA-ILD remains obscure, the current data indicate that neutrophils play a role in the progression of RA-ILD through disease-specific inflammatory NETs [35]. Our study revealed that the levels of MPO-DNA in the serum and BALF, the levels of the PADI4 and Cit-H3 proteins in the lungs, and the mRNA levels of *padi4* in the lungs were significantly elevated in SKG mice following exposure to ZYM. However, no significant difference was observed in the lung MPO-DNA levels of SKG mice exposed to ZYM. This lack of difference may be attributed to the interaction between MPO and DNA within neutrophils during the process of NET formation, which ultimately leads to the extracellular release of the MPO‒DNA complex [36]. Additionally, our research demonstrated that plasma MPO-DNA, Cit-H3, and cell-free DNA were elevated in RA-ILD patients compared with HCs.

The formation of NETs is a complex process involving multiple molecular mechanisms and signaling pathways. In this study, PMA- and RA-ILD patient serum-induced HL-60 cell models were used to explore the molecular mechanism of NET formation. However, research has demonstrated that only 10% of dHL-60 cells can release NET-like structures after stimulation with a variety of compounds [37]. Our study, as well as a previous study [38], revealed that HL-60 cells with fewer passages were more likely to release NETs after PMA induction. Our present results show that NR4A3 inhibits NET formation induced by RA-ILD patient serum. This observation is in agreement with the findings of a recent study suggesting that NR4A3 plays a role in diabetes-induced atrial cardiomyopathy by maintaining mitochondrial energy metabolism and reducing oxidative stress [17]. This conclusion will be further validated in primary neutrophils in our future studies.

Previous studies have demonstrated that NET components such as DNA, MPO, NE, and histones release cytokines that lead to inflammation, epithelial‒mesenchymal transition (EMT), epithelial damage, fibroblast activation, and fibroblast‒myofibroblast transition, all of which promote the progression of lung fibrosis [39]. In line with these findings, our study demonstrated that NETs secreted from HL-60 cells promoted the transformation of MRC-5 cells into myofibroblasts. Recent research has demonstrated that neutrophils release NETs containing proteins such as NE, cathelicidin (LL-37), PADI4, and tissue factor (TF), which directly influence fibroblast behavior [35, 40]. NE induces fibroblast transdifferentiation into myofibroblasts, enhancing contractility and extracellular matrix (ECM) remodeling. LL-37 promotes collagen production via formyl peptide receptor-like 1 (FPRL1) receptor binding, whereas PADI4-mediated citrullination of ECM proteins disrupts integrin-ECM interactions, leading to fibroblast migration and resistance to apoptosis. TF activation of fibroblasts via proteinase-activated receptor 2 (PAR-2) signaling further stimulates ECM production and proliferation. These processes, in conjunction with the persistent inflammatory environment induced by NETs, create a positive feedback loop that amplifies fibrosis, impairs wound resolution, and contributes to the progressive loss of lung architecture and function [40]. In future studies, we will utilize single-cell data to identify fibroblast receptors that interact with NET components and assess their role in regulating fibroblast-to-myofibroblast differentiation.

This study revealed that plasma MPO-DNA and Cit-H3 levels were greater in RA-NSIP and RA-UIP patients than in HCs. Additionally, the ROC curves revealed that RF, anti-CCP antibody, and MPO-DNA were helpful in diagnosing NSIP in RA-ILD patients, whereas RF, anti-CCP antibody, and Cit-H3 were useful in diagnosing UIP in RA-ILD patients. Most studies report NSIP and UIP as the most common patterns, with the coexistence of UIP and NSIP being common in RA-ILD [41]. Recent research has proposed a mechanism of arthritis-onset RA-ILD (RA-NSIP): RF directly contributes to the pathogenesis of RA by potentiating a cycle of immune complex formation and complement fixation, which leads to additional autoantibody production (e.g., anti-CCP antibodies) affecting joints and subsequently the lungs [42, 43]. ILD-onset RA-ILD (RA-UIP) involves an autoimmune response against citrullinated proteins in the lung, resulting in high levels of anti-CCP antibody, which subsequently promotes arthritis [43]. Anti-CCP-positive ILD patients with positive RF are at increased risk of developing RA [44]. These findings suggest that the mechanisms may differ between radiological subtypes of RA-ILD but are likely linked to RF and anti-CCP antibodies. However, the titers of these two antibodies undoubtedly differ as initiating factors for the development of the RA-NSIP and RA-UIP subtypes, which may account for the correlation between different autoantibody markers and distinct patterns of RA-ILD. In this study, strong correlations between RF and anti-CCP antibody were observed in both the MPO-DNA-positive and MPO-DNA-negative RA-NSIP groups, as well as in the Cit-H3-positive and Cit-H3-positive RA-UIP groups. These findings suggest that the mechanisms driving NET formation may differ between radiological subtypes of RA-ILD. Somewhat unexpectedly, cell-free DNA did not correlate well with the RF and anti-CCP antibodies in RA-UIP patients, possibly because cell-free DNA is not a highly specific marker for NETs. This finding may be attributed to the fact that neutrophils are relatively short-lived cells that may experience cell death through many pathways, including apoptosis, necrosis, pyroptosis, and NETosis. Markers such as cell-free DNA may therefore also be produced by neutrophil death, which is independent of NETosis [45, 46]. Therefore, we will expand our sample size in future studies and combine the detection of MPO-DNA, Cit-H3, RF, and anti-CCP antibodies for the differential diagnosis of clinical RA-NSIP and RA-UIP patients, which may result in personalized treatment in clinical settings. Additionally, neutrophils from patients with RA-UIP exhibited a significantly greater capacity to form NETs and induce the differentiation of MRC-5 cells into myofibroblasts. This finding could be attributed to the fact that UIP is the radiological form of ILD related to IPF in clinical settings, where fibroblasts are the final effector cells involved in the progression of IPF. In contrast, RA-NSIP was characterized by the predominance of ground-glass opacity (GGO), possible visible subpleural sparing and possible fine reticulation with minor or no honeycombing [47]. Research has shown that nintedanib administration may effectively complement activation and NET formation in patients with RA-ILD [35]. In light of our findings, we hypothesize that nintedanib may be more effective in patients with RA-UIP. In future studies, we plan to retrospectively collect clinical data from RA-ILD patients and prospectively include RA-ILD patients to investigate the correlation between nintedanib treatment and radiological subtypes of RA-ILD.

The current study was subject to several limitations. First, this study was conducted on a small sample at a single center, and follow-up data were lacking. Future studies with a larger patient cohort and long-term follow-up using RA-ILD indices are needed. Second, although plasma MPO-DNA and Cit-H3 levels are elevated in patients diagnosed with RA-NSIP and RA-UIP, the lack of RA-ILD lung biopsies and BALF limits the ability to assess the correlation between NETs and local pathological changes within the lungs. Third, the underlying molecular mechanisms by which NR4A3 leads to a reduction in the formation of NETs remain largely unknown. On the basis of reports in the literature on NR4A3, we hypothesize that NR4A3 may indirectly inhibit the formation of NETs by downregulating the activity of NOX2 and further reducing the generation of ROS. Finally, the NET and RA-ILD data presented are all associative, not causative. In future research, we will aim to deplete PMNs to investigate their potential role in improving ILD.

## Conclusions

Despite some limitations, our study clearly shows that the pathological characteristics of ZYM-treated SKG mice closely mimic those observed in RA-ILD. In addition, the expression of NR4A3, a key regulator of NET formation and fibroblast-to-myofibroblasts transformation, plays a crucial role in the development of ILD in RA patients. Furthermore, combining MPO-DNA, RF, and anti-CCP enhances diagnostic accuracy for identifying NSIP in RA-ILD patients, while combining Cit-H3, RF, and anti-CCP improves diagnosis of UIP. Finally, neutrophils from RA-UIP are more prone to NET formation and induce the differentiation of MRC-5 cells into myofibroblasts. Pharmacotherapeutic targeting of NETs or the use of NR4A3 antagonists might be promising therapeutic approaches for attenuating RA-ILD, particularly in individuals with the UIP pattern.

## Electronic supplementary material

Below is the link to the electronic supplementary material.

Supplementary Material 1

Supplementary Material 2

Supplementary Material 3

Supplementary Material 4

Supplementary Material 5

## Data Availability

The transcription profile dataset of lung tissues in RA-UIP, IPF-UIP and non-UIP control groups (GSE199152) was obtained from NCBI GEO database .

## Abbreviations

ACPA: Anticitrullinate protein antibodies
ACR: American College of Rheumatology
ARRDC2: Arrestin domain containing 2
AUC: Area under the curve
Cit-H3: Citrullinated histon3
C3: Complement C3
FC: Fold change
FOS: Fos proto-oncogene
HRCT: High-resolution computed tomography
ILD: Interstitial lung disease
IPF: Idiopathic pulmonary fibrosis
MPO: Myeloperoxidase
NE: Neutrophil elastase
NETs: Neutrophil extracellular traps
NR4A3: Nuclear receptor subfamily 4 group A member 3
NSIP: Nonspecific interstitial pneumonia
PADI4: Protein arginine deiminase
PFTs: Pulmonary function tests
RA: Rheumatoid arthritis
RF: Rheumatoid factor
ROC: Receiver operating characteristic
ROS: Reactive oxygen species
SMAD6: SMAD family member 6
