# Supplementary figures and images for: Neutrophil extracellular traps (NETs) are increased in rheumatoid arthritis-associated interstitial lung disease
Source: Respir Res. 2025 Jan 22;26:33. doi: 10.1186/s12931-025-03111-1 (PMC11756115; doi:10.1186/s12931-025-03111-1)

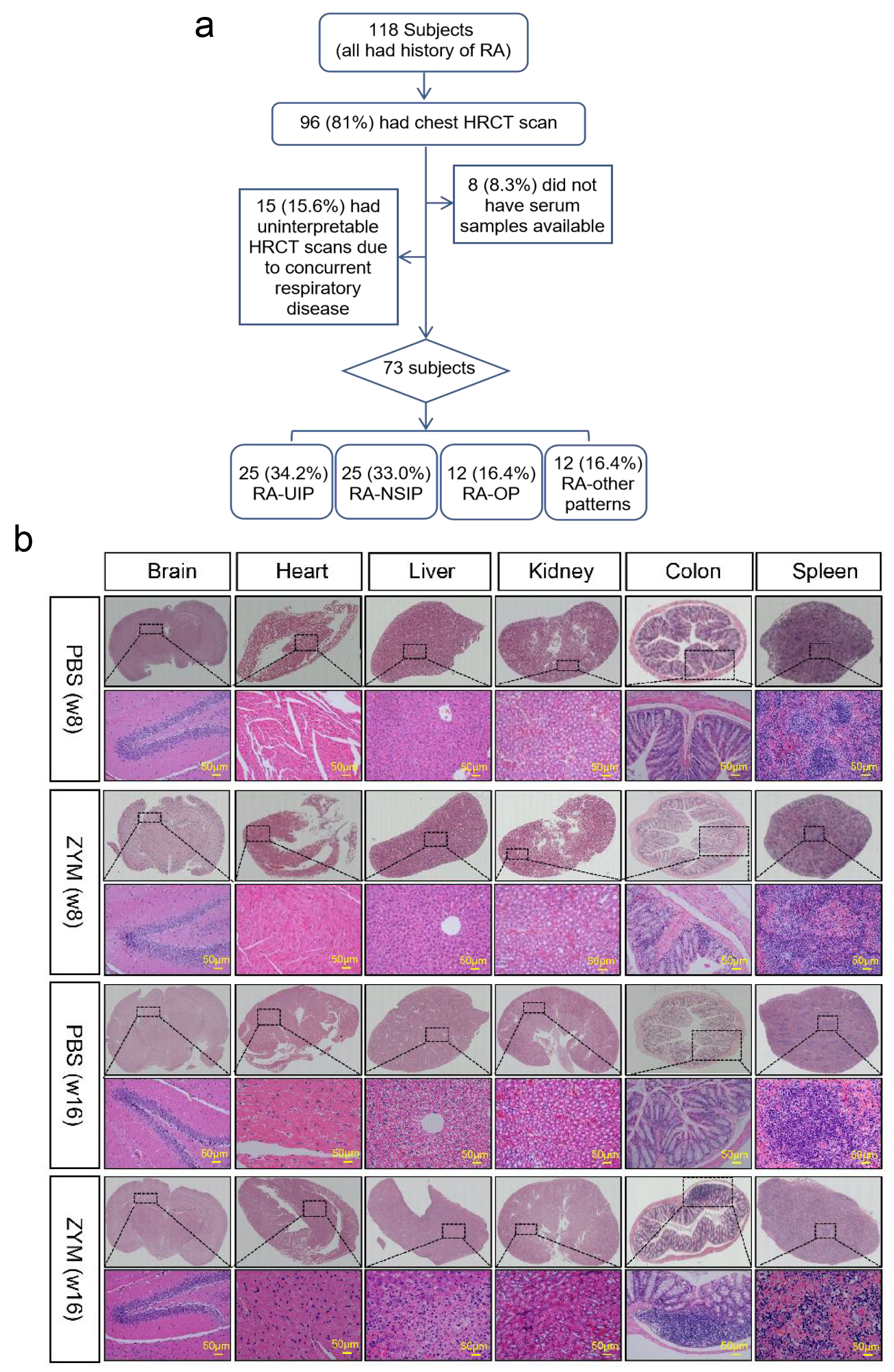

Supplement: Supplementary file 1 — Supplementary Material 1 [file 12931_2025_3111_MOESM1_ESM.tif]

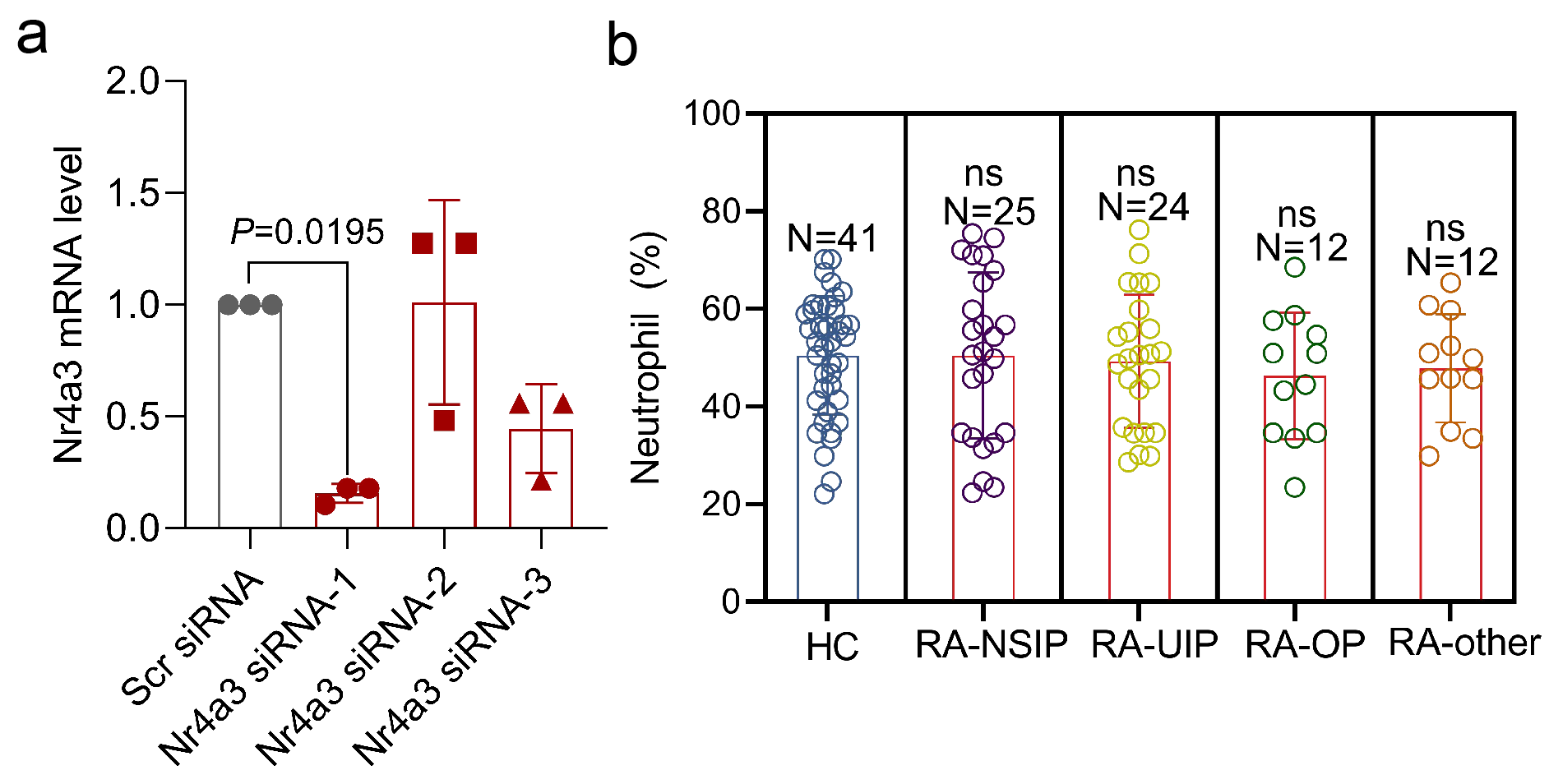

Supplement: Supplementary file 2 — Supplementary Material 2 [file 12931_2025_3111_MOESM2_ESM.tif]
