# Supplementary material for: Neutrophil extracellular traps (NETs) are increased in rheumatoid arthritis-associated interstitial lung disease
Source: Respir Res. 2025 Jan 22;26:33. doi: 10.1186/s12931-025-03111-1 (PMC11756115; doi:10.1186/s12931-025-03111-1)
Supplement: Supplementary file 3 — Supplementary Material 3 [file 12931_2025_3111_MOESM3_ESM.docx]

**Gels and blots (Figure 1p)**

**Protein marker:** **200, 140,110, 75, 55, 42, 30, 23, 18, 10**

**TGF-β1 (44kDa)**

**PBS (w16) ZYM (w16)**


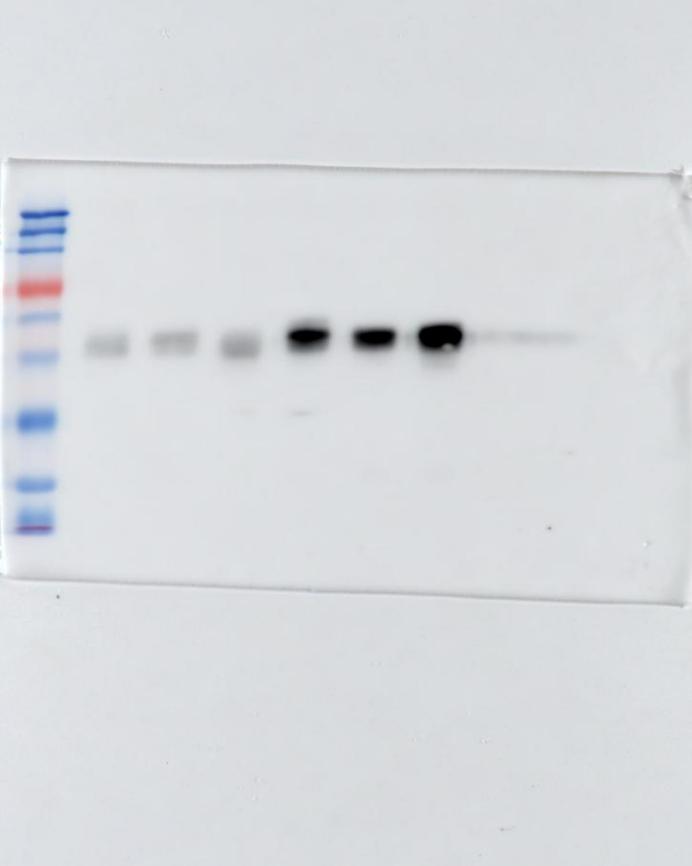


10

75

**α-SMA (42kDa)**

**PBS (w16) ZYM (w16)**


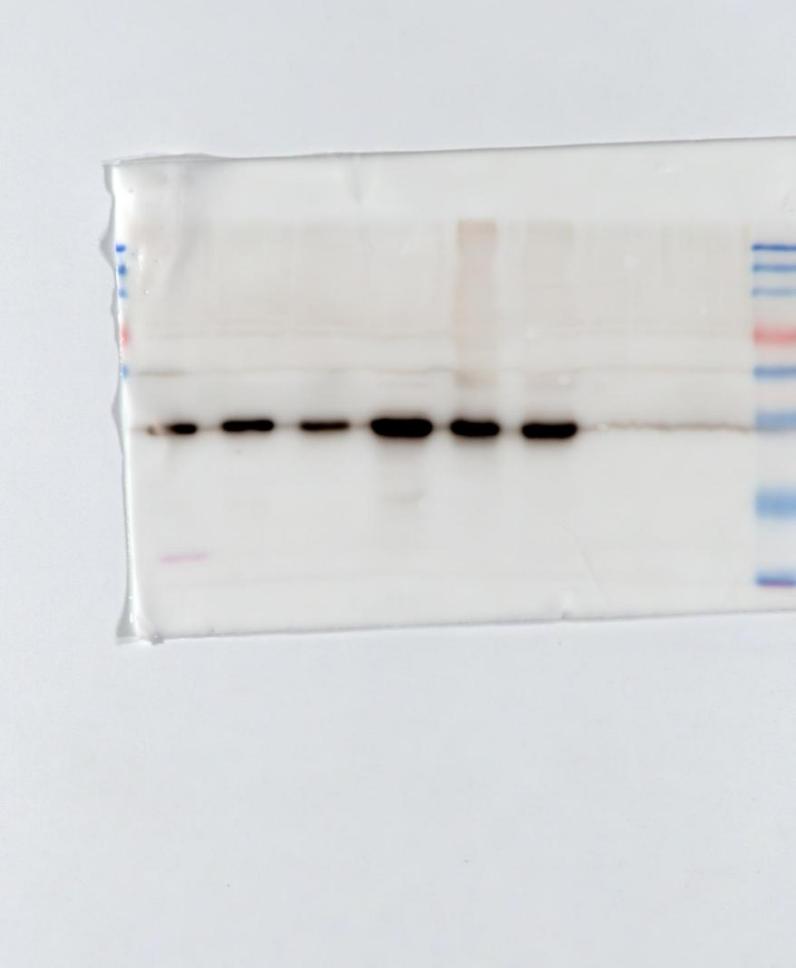


10

75

**GAPDH (36kDa)**

**PBS (w16) ZYM (w16)**


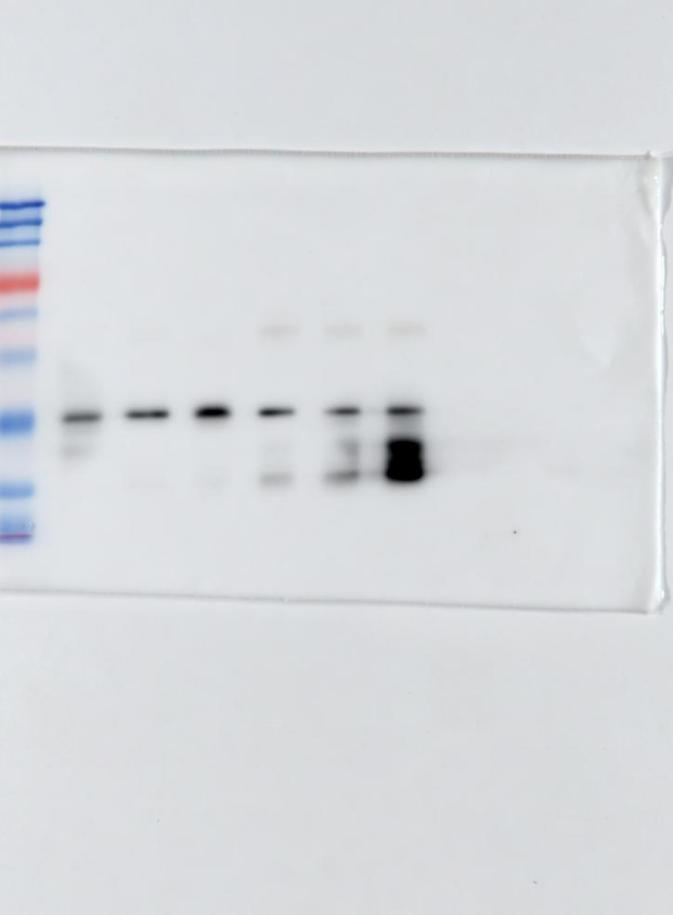


10

75

**Gels and blots (Figure 3j)**

**Protein marker:** **200, 140,110, 75, 55, 42, 30, 23, 18, 10**

**PADI4 (67kDa)**

**PBS (w16) ZYM (w16)**

**
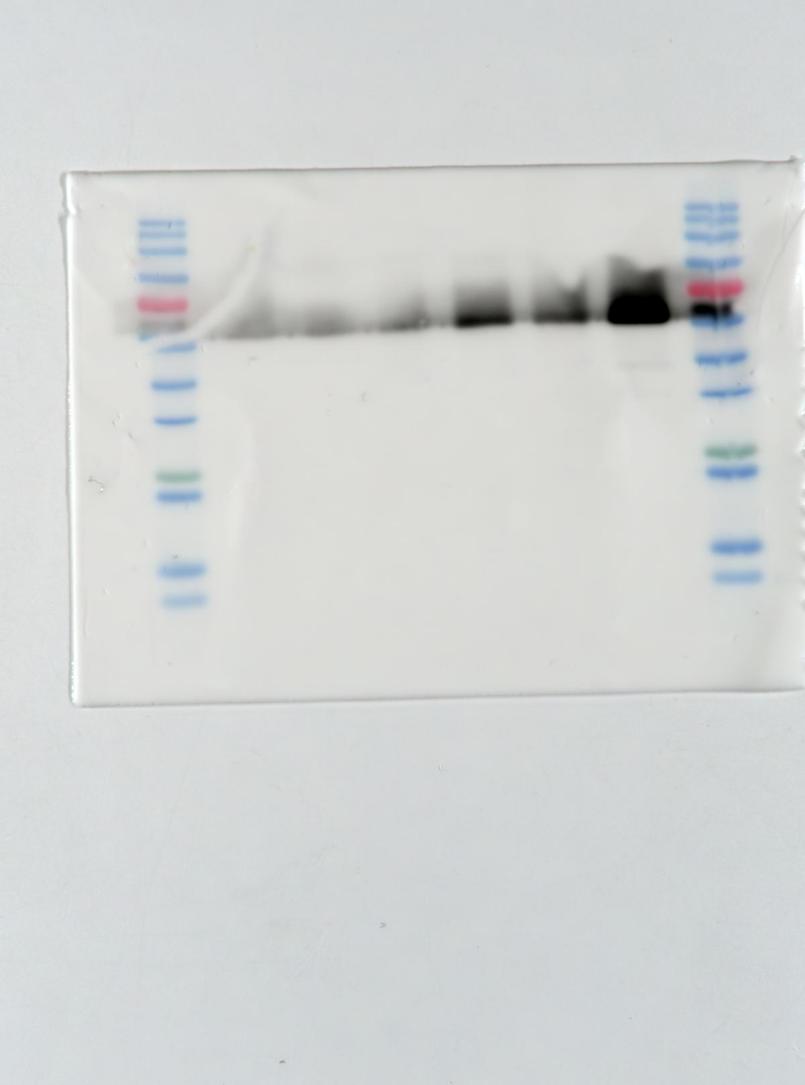
**

75

**Cit-H3 (17kDa)**

**PBS (w16) ZYM (w16)**


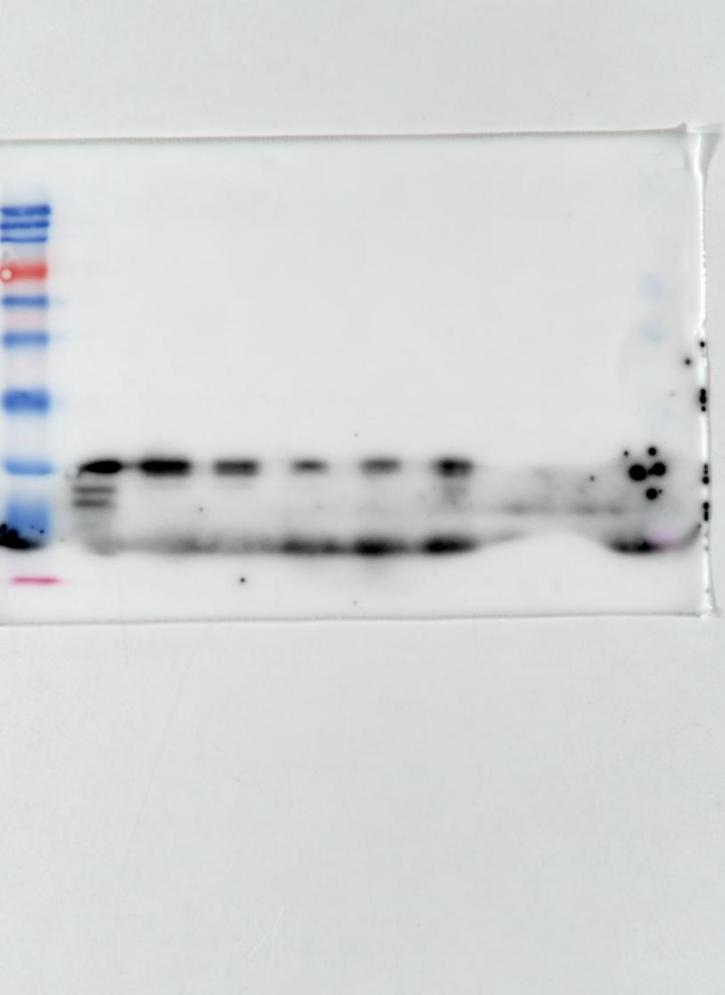


10

75

**GAPDH (36kDa)**

**PBS (w16) ZYM (w16)**


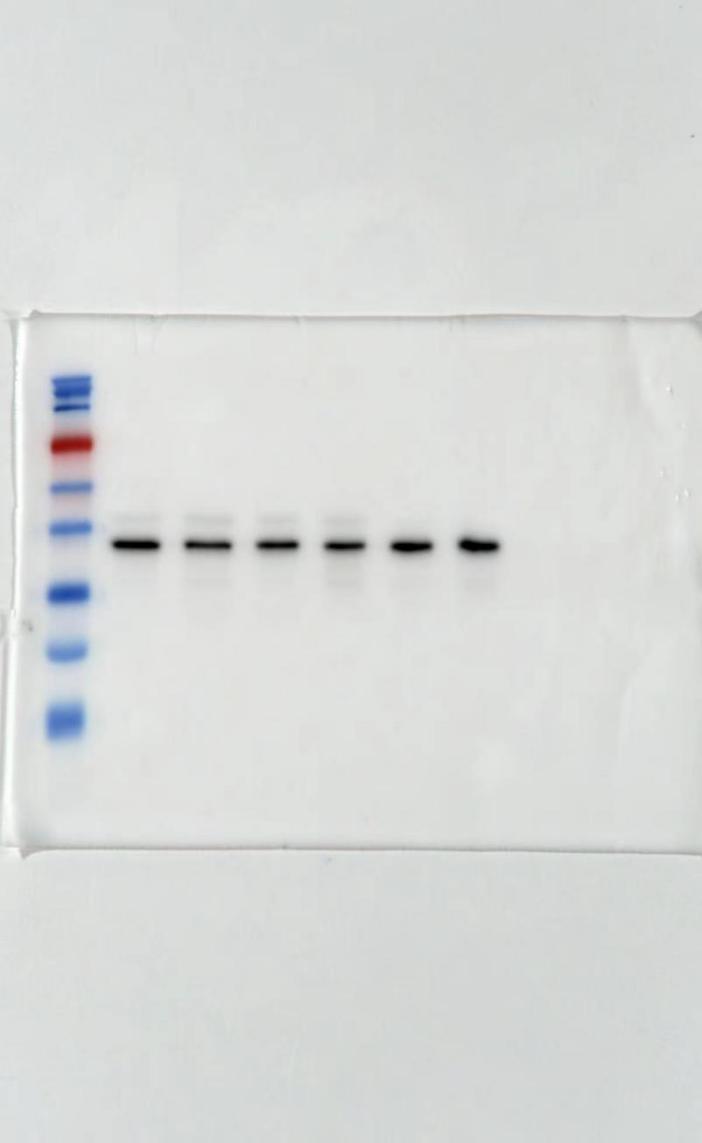


75
