# Supplementary material for: Neutrophil extracellular traps (NETs) are increased in rheumatoid arthritis-associated interstitial lung disease
Source: Respir Res. 2025 Jan 22;26:33. doi: 10.1186/s12931-025-03111-1 (PMC11756115; doi:10.1186/s12931-025-03111-1)
Supplement: Supplementary file 4 — Supplementary Material 4 [file 12931_2025_3111_MOESM4_ESM.doc]

**Supplementary Table S1. Primer used in the present study.**

| **Gene** | **Forward primers (5’-3’)** | **Reverse primers (5’-3’)** |
| --- | --- | --- |
| Gapdh | TGTGTCCGTCGTGGATCTGA | TTGCTGTTGAAGTCGCAGGAG |
| Padi4 | CTCTCCAGGAGTCATCGTAG | CCAACACCAGCTGATACTTT |

**Supplementary Table S2. List of primary antibodies employed in this study.**

| **Protein** | **Vendor** | **Product no.** | **Type** | **Dilution** |
| --- | --- | --- | --- | --- |
| PADI4 | Proteintech | 17373-1-AP | Rabbit Polyclonal | 1:400 |
| Cit-H3 | abmart | #P63763-2B3 | Rabbit Polyclonal | 1:300 |
| Collagen1 | Abcam | ab138492 | Rabbit Polyclonal | 1:1000 |
| α-SMA | Proteintech | CL488-14395 | Rabbit Polyclonal | 1:400 |
| TGF-β1 | Abcam | Ab27969 | Rabbit Polyclonal | 1:200 |
| LY6G | Proteintech | 65078-1-lg | Mouse Monoclonal | 1:200 |

**Supplementary Table S3. Baseline characteristics of RA-ILD (n=73), and HC (n=41).**

|  | **Cohort, No.(%)** | |  |
| --- | --- | --- | --- |
| **Variable** | **RA-ILD (n=73)** | **HC (n=41)** | **P Value** |
| **Demographic features** |  |  |  |
| Age, years | 59.7±7.0 | 54.2±1.1 | 0.56 |
| Sex |  |  |  |
| Male | 45 (61.6%) | 25 (60.1%) | 0.81 |
| Female | 28 (38.4%) | 16 (39.9%) |
| Current smoker | 56 (76.7%) | 30 (73.2%) | 0.63 |
| Clinical features |  |  |  |
| RA duration, years | 9.1±0.9 | - | - |
| DAS28 score | 2.8±0.2 | - | - |
| ILD duration, years | 2.2±0.4 | - | - |
| **Serologic features** |  |  |  |
| AKA (+) | 60 (82.1%) | - | - |
| RF (+) | 69 (94.5%) | - | - |
| Anti-CCP (+) | 68 (93.2%) | - | - |
| ESR (mm/h) | 106.6±7.5 | - | - |
| CRP (mg/dl) | 97.0±8.4 | - | - |
| **ILD pattern** |  |  |  |
| UIP | 24 (33.0%) | - | - |
| NSIP | 25 (34.2%) | - | - |
| OP | 12 (16.4%) | - | - |
| Others | 12 (16.4%) | - | - |

RA: rheumatoid arthritis; ILD: interstitial lung disease; RF: rheumatoid factor; CCP: cyclic citrullinated; ESR: erythrocyte sedimentation rate; CRP: c-reactive protein; UIP: usual interstitial pneumonia; NSIP: nonspecific interstitial pneumonia; OP: organizing pneumonia.
